# The impact of PET/CT and brain MRI for metastasis detection among patients with clinical T1-category lung cancer: Findings from a large-scale cohort study

**DOI:** 10.1007/s00259-024-06740-8

**Published:** 2024-05-09

**Authors:** Yi Feng, Bo Cheng, Shuting Zhan, Haiping Liu, Jianfu Li, Peiling Chen, Zixun Wang, Xiaoyan Huang, Xiuxia Fu, Wenjun Ye, Runchen Wang, Qixia Wang, Yang Xiang, Huiting Wang, Feng Zhu, Xin Zheng, Wenhai Fu, Guodong Hu, Zhuxing Chen, Jianxing He, Wenhua Liang

**Affiliations:** 1 Department of Thoracic Surgery and Oncology, China State Key Laboratory of Respiratory Disease & National Clinical Research Center for Respiratory Disease, the First Affiliated Hospital of Guangzhou Medical University, Guangzhou, 510120 China; 2 Guangzhou Institute of Respiratory Health, Guangzhou, 510120 China; 3 PET/CT Center, The First Affiliated Hospital of Guangzhou Medical University, Guangzhou, China; 4 Nanshan School, Guangzhou Medical University, Jingxiu Road, Panyu District, Guangzhou, 511436 China; 5 The Radiology Department of the First Affiliated Hospital of Guangzhou Medical University, Guangzhou, 510120 China; 6 Detroit Medical Center Sinai-Grace Hospital, Internal Medicine Department, 6071 Outer Dr W, Detroit, MI 48235 USA; 7 Department of Respiratory and Critical Care Medicine, The Tenth Affiliated Hospital of Southern Medical University, Dongguan, Guangdong 523108 China; 8 Pulmonary Nodule Surgical Department, The First People’s Hospital of Foshan, Foshan, 528000 China; 9 Department of Thoracic Surgery, NANFANG Hospital of Southern Medical University, Guangzhou, China; 10 Department of Oncology Medical Center, The First People’s Hospital of Zhaoqing, Zhaoqing City, Guangdong Province China

**Keywords:** Clinical T1-category Lung Cancer, PET/CT, Brain MRI, Metastasis

## Abstract

**Purpose:**

[^18^F]-FDG PET/CT and brain MRI are common approaches to detect metastasis in patients of lung cancer. Current guidelines for the use of PET/CT and MRI in clinical T1-category lung cancer lack risk-based stratification and require optimization. This study stratified patients based on metastatic risk in terms of the lesions' size and morphological characteristics.

**Methods:**

The detection rate of metastasis was measured in different sizes and morphological characteristics (solid and sub-solid) of tumors. To confirm the cut-off value for discriminating metastasis and overall survival (OS) prediction, the receiver operating characteristic (ROC) analysis was performed based on PET/CT metabolic parameters (SUVmax/SUVmean/SULpeak/MTV/TLG), followed by Kaplan–Meier analysis for survival in post-operation patients with and without PET/CT plus MRI.

**Results:**

2,298 patients were included. No metastasis was observed in patients with solid nodules < 8.0 mm and sub-solid nodules < 10.0 mm. The cut-off of PET/CT metabolic parameters on discriminating metastasis were 1.09 (SUVmax), 0.26 (SUVmean), 0.31 (SULpeak), 0.55 (MTV), and 0.81 (TLG), respectively. Patients undergoing PET/CT plus MRI exhibited longer OS compared to those who did not receive it in solid nodules ≥ 8.0 mm & sub-solid nodules ≥ 10.0 mm (HR, 0.44; p < 0.001); in solid nodules ≥ 8.0 mm (HR, 0.12; p<0.001) and in sub-solid nodules ≥ 10.0 mm (HR; 0.61; p=0.075), respectively. Compared to patients with metabolic parameters lower than cut-off values, patients with higher metabolic parameters displayed shorter OS: SUVmax (HR, 12.94; p < 0.001), SUVmean (HR, 11.33; p <0.001), SULpeak (HR, 9.65; p < 0.001), MTV (HR, 9.16; p = 0.031), and TLG (HR, 12.06; p < 0.001).

**Conclusion:**

The necessity of PET/CT and MRI should be cautiously evaluated in patients with solid nodules < 8.0 mm and sub-solid nodules < 10.0 mm, however, these examinations remained essential and beneficial for patients with solid nodules ≥ 8.0 mm and sub-solid nodules ≥ 10.0 mm.

**Supplementary Information:**

The online version contains supplementary material available at 10.1007/s00259-024-06740-8.

## Introduction

Lung cancer remains the predominant cause of cancer-related mortality worldwide and ranks the most common cancer globally [1]. The advent of widespread lung cancer screening has markedly elevated the detection rates of early-stage lung cancer [2, 3]. Early-stage lung cancer typically manifests as pulmonary nodules on computed tomography (CT) scan, appearing as small size (≤30 mm), focal and discrete radiographic density surrounded by lung parenchyma [4].

[^18^F]-fluorodeoxyglucose ([^18^F]-FDG) Positron Emission Tomography/Computed Tomography (PET/CT) is a common approach in the metastases detection and staging of lung cancer, and provides useful data for the characterization of morphologically indeterminate pulmonary nodules and has been widely adopted in clinical practice [5]. The deficiency of PET/CT in cerebral imaging necessitates the complementary use of contrast-enhanced brain MRI for thorough assessment of potential brain metastases. According to the National Comprehensive Cancer Network (NCCN) Clinical Practice Guidelines, PET/CT is recommended in all confirmed cases of lung cancer (category 2A recommendation), irrespective of tumor size [6, 7]. The European Society of Medical Oncology (ESMO) Clinical Practice Guidelines endorses preoperative PET/CT in lung cancer to rule out mediastinal lymph node metastasis, though it stops short of specifying a nodule size threshold for this recommendation [8]. MRI is recommended for all lung cancer patients except those with stage IA peripheral non-small cell lung cancer (NSCLC) [6, 7]. Previous studies have shown that the metastatic potential of lung cancer correlates with its invasiveness and varies across tumors of different sizes and compositions (solid or ground glass nodules) [4, 9]. Consequently, in early-stage lung cancer patients harboring low-invasive lesions of small size or ground-glass opacity (GGO), the utilization of PET/CT and MRI could be exempted due to their weak metastatic risk. Therefore, a more definite recommendation involved in lesions’ diameter and density for PET/CT and MRI examinations in these early-stage lung cancer patients is warranted [10, 11].

In this study, we aimed to stratify patients based on metastatic risk and optimize existing guidelines for the application of PET/CT and MRI in the management of clinical T1-category lung cancer patients manifesting as solid and sub-solid nodules.

## Material and methods

This was a single-center, retrospective study utilizing a large-scale prospectively maintained cohort. Approval was obtained from institutional review board, and the requirement for informed consent was waived, considering its retrospective nature.

### Study design and participants

Clinical-pathological data and survival information were retrieved from the database of our hospital. All pathologically diagnosed (via surgery or biopsy) lung cancer patients who underwent both PET/CT and contrast-enhanced brain MRI examinations were included between January 2009 and July 2022. Exclusion criteria were as follows: (1) patients with incomplete clinical information; (2) accompanied with other system malignancies history; (3) clinical T2, T3, or T4-categories lung cancers; (4) multiple primary lung cancer (MPLC); and (5) those who received anti-cancer therapy prior to the PET/CT plus MRI examination.

### Image acquisition protocol

Scanning procedures were performed using a GE Discovery ST 8 scanner (GE Healthcare Lunar, Wisconsin, USA), employing [^18^F]-FDG as the radiotracer with excellent chemical identity and purity (> 95%). The patients adhered to a 6-h fasting period prior to data acquisition. Height and weight measurements, along with fasting blood glucose levels were obtained, and maintained at < 8.0 mmol/L. Patients received an intravenous injection of 3.7 MBq/kg (0.1 mCi/kg) [^18^F]-FDG via the dorsal hand vein in a resting state. PET/CT imaging commenced 60–90 min post-injection, with patients resting in a dimly lit room to reduce external stimuli. The patients were prepared for the PET/CT examination in a supine position. A multi-slice spiral CT scan was performed first, covering a wide range from the head to the feet. The following scan parameters were utilized: tube voltage of 140 kV, tube current of 150 mA, pitch of 0.875, field of view (FOV) of 50 cm, slice thickness of 3.75 mm and a matrix 512 × 512. The PET scanning was performed in the same scanning range, using 2D acquisition mode and matrix of 128 × 128. The acquisition time was 3 min/bed position, and 6–7 bed position were collected. Bayesian penalized likelihood reconstruction algorithms were applied to reconstruct PET/CT images in 3D, optimizing image resolution and quality. Simultaneous acquisition and fusion of PET and CT images were performed automatically, utilizing CT image data for attenuation correction of PET images. A thin-slice CT scan was conducted during a breath-hold on all lung nodules with a slice thickness of 1.25 mm, ensuring optimal reconstruction and reducing the impact of respiratory motion artifacts on PET image quality.

3.0-T MRI scanner (Philips Achieva) was employed for contrast-enhanced brain MRI with the following parameters: axial T2WI (repetition time (TR), 3000 ms; echo time (TE), 80 ms; the number of excitations (NEX), 1; slice thickness, 5 mm, FOV, 230 × 230 mm; matrix 720 × 720), intravenous administration of contrast agent Gd-DTPA-BMA (TR, 600 ms; TE, 28 ms; NEX, 1; slice thickness, 5 mm; FOV, 240 × 240; matrix 864 × 864) for axial, coronal and/or sagittal T1WI. DWI sequence (TR 2989 ms; TE, 90 ms; NEX, 1; slice thickness, 5 mm; FOV, 230 × 230 mm; matrix, 256 × 256); and MRA (TR, 23 ms; TE, 3.45 ms; NEX, 1; slice thickness, 1.4 mm; FOV, 180 × 180 mm; matrix, 480 × 480).

### Image analysis

PET/CT images were independently evaluated by two experienced nuclear medicine physicians, who were apprised of the initial clinical data but remained blinded to the histologic outcomes and MRI findings. Any diagnostic discrepancies were resolved through a consensus approach. The analysis focused on semi-quantitative parameters of all discernible lesions. Utilizing a 3D regional growth algorithm, lesions were automatically segmented and delineated on axial, coronal, and sagittal planes to ensure precise and objective assessment. Metabolic parameters such as maximum standardized uptake value (SUVmax), mean standardized uptake value (SUVmean), standardized uptake values corrected for lean body mass (SULpeak), metabolic tumor volume (MTV), total lesion glycolysis (TLG = SUVmean×MTV) were calculated in same mode. We used a fixed threshold of 40% SUVmax for tumor delineation.

### Outcomes

Clinical data encompassed variates such as gender, age, nodule diameter, location, histologic findings, and the presence of epidermal growth factor receptor (EGFR) mutations. Included patients (lesions) were assessed radiologically based on the maximum dimensions and classified as solid and sub-solid [12]. The staging of lymph node and distant metastases were clinically performed and involved collaboration between a thoracic radiologist (X.Y.H., with a decade of experience in cardiopulmonary imaging) and a medical student (B.C., with five years of experience in pulmonary imaging diagnostics), who reached an in accordance with the 8th edition of the Tumor Node Metastasis (TNM) staging system [13]. The detection rate of metastasis was herein defined as the percentage of clinical T1-category lung cancer patients who developed metastases. The diameter cut-off that corresponded to 0% detection rates of metastasis were observed.

The survival of enrolled patients who received surgical resection of tumor was analyzed, and a cohort of clinical T1-category post-operation patients who underwent CT only (or plus MRI) was set as control group, and the inclusion and exclusion criteria were the same for the PET/CT plus MRI. The primary objective was to compare the 5-year overall survival (OS, time from pathological diagnosis to death from lung cancer) in patients without baseline metastasis between those who received PET/CT plus MRI and those who underwent CT only (or plus MRI), furthermore, OS among PET/CT plus MRI, CT plus MRI, and CT only were compared.

### Statistical analysis

Patient demographics and tumor characteristics, including age and nodule diameter, were quantitatively summarized using median values and interquartile ranges (IQR). Categorical variables such as gender, lesion locations, histologic findings, and the presence of EGFR mutations were reported as percentages. Group comparisons were conducted using the Chi-square test, Fisher’s exact test, or Mann–Whitney U test, as appropriate. Receiver operating characteristic (ROC) analysis was performed to evaluate the diagnostic efficacy of PET/CT based on the binary variable as 5-year OS, and areas under the ROC curve (AUC) were calculated to confirm the optimal cut-off of PET/CT metabolic parameters that maximize specificity for metastasis detection (corresponding to the cut-off of 0% detection rate of metastasis) and OS prediction based on Youden's index. The OS was estimated using the Kaplan–Meier (K-M) methodology, and data for patients who were alive or lost to follow-up were censored at their last known contact (time cut-off: April 20, 2023). The stratified log-rank test was used to assess differences in OS between groups. For the assessment of hazard ratios (HRs) and their corresponding two-sided 95% confidence intervals (CIs), we employed the Cox proportional hazards model, incorporating Efron’s method for tie-handling [14]. Finally, the propensity-score matching (PSM) procedure was applied to balance baseline covariates and minimize potential confounding factors between these groups in post-operation [15], matching covariates encompassed the following factors: gender, age, nodule diameter, location, pathologic risk factors (pleural invasion, vascular cancer embolus, spread through air spaces, nerve invasion, bronchial invasion, and vascular invasion) and histologic findings. All matching covariates were matched at a ratio of 1:1 and a caliper of 0.02. Statistical significance was set at a p-value of less than 0.05, and all analyses were performed using Microsoft Excel (Version 2016) and R Studio (version 4.2.2).

## Results

### Clinical characteristics

**Fig. 1:**
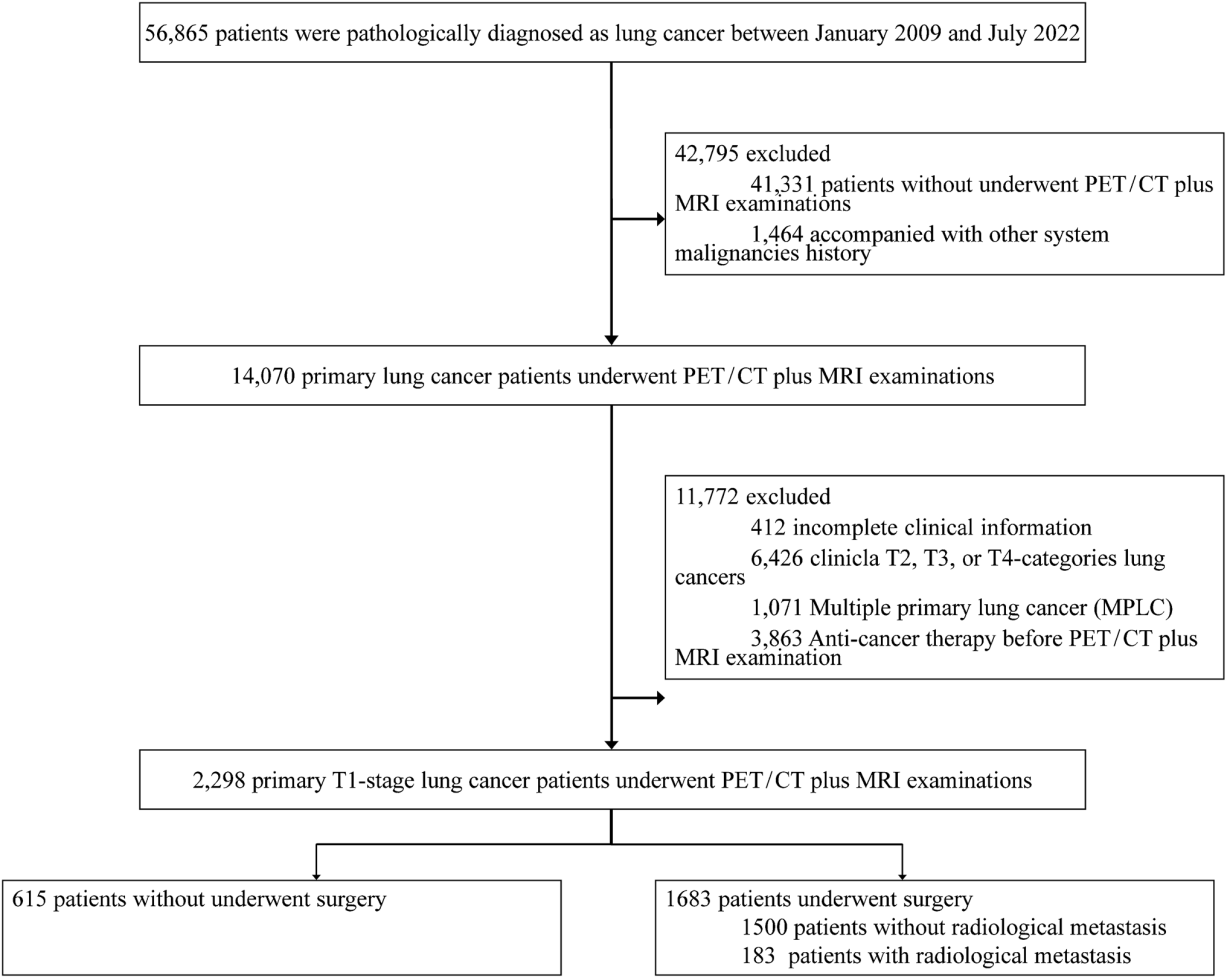
Flowcharts depicting criteria for inclusion and exclusion of study participants

Figure 1 shows the patient inclusion and exclusion flowchart. During the period from January 2009 to July 2022, a total of 56,865 lung cancer patients were initially considered for inclusion. Of these, 27.3% (15,534 patients) underwent both PET/CT and MRI examinations. Exclusions were subsequent made based on the following criteria: accompanied with other system malignancies history (1,464 patients); incomplete clinical information (412 patients); clinical T2, T3, or T4-categories lung cancers (6,426 patients); multiple primary lung cancer (1,071 patients); anti-cancer therapy before PET/CT examination (3,863 patients). Finally, the study cohort was comprised of 2,298 clinical T1-category lung cancer patients who underwent PET/CT plus MRI (Fig. 1). Of these, 1,017 patients (44.3%) were female, and the median age was 60 years (IQR, 52–66 years). The median nodules diameter was 20.0 mm (IQR, 15.0–25.0 mm), of which 653 (28.4%) patients with solid nodules (Table 1). Anatomically, the nodules were distributed as follows: Left upper lobe (LUL) 27.4% (629 patients), Left lower lobe (LLL) 13.4% (308 patients), Right upper lobe (RUL) 34.5% (792 patients), Right middle lobe (RML) 6.9% (158 patients), Right lower lobe (RLL) 17.6% (404 patients), not available (NA) 0.3% (7 patients). The histological findings included lung adenocarcinoma (LUAD) 82.3% (1,892 patients), lung squamous cell carcinoma (LUSC) 5.3% (121 patients), small cell lung cancer (SCLC) 2.2% (51 patients), and other types 10.2% (234 patients). Among these patients, 529 (23.0%) were tested for EGFR mutations, of which 247 (46.7%) were positive. Among the cohort, 457 male patients (35.7%) developed metastases (p < 0.001), results showed that patients with metastases were of similar median age (60 years; IQR, 52–66 years; p = 0.866) and EGFR mutation (p = 0.092) as those without metastases. Significant differences were observed in nodule diameter (median diameter: 23.0 mm, IQR, 20.0–27.0 mm), nodule location (p = 0.002), histological findings (p < 0.001), SUVmax (p < 0.001), SUVmean (p < 0.001), SULpeak (p = 0.008), MTV (p = 0.003) and TLG (p < 0.001). The demographic and clinical-pathological features of lung cancer, categorized by solid and sub-solid nodules, detailed in Table 1.

**Table 1:** The demographic and clinical-pathological features of T1-category lung cancer patients with pulmonary nodules

		**All**			**Solid**			**Sub-solid**		
		Metastasis	No metastasis		Metastasis	No metastasis		Metastasis	No metastasis	
Gender				P value			P value			P value
	Male	457 (35.7%)	824 (64.3%)	< 0.001	198 (47.5%)	219 (52.5%)	0.345	259 (30.0%)	605 (70.0%)	< 0.001
	Female	229 (22.5%)	788 (77.5%)		103 (43.6%)	133 (56.4%)		126 (16.1%)	655 (83.9%)	
Age (Year)										
	Median (IQR)	60 (52–66)	59 (51–66)	0.866	61 (53–66)	62 (54–68)	0.093	59 (51–66)	59 (51–66)	0.639
Nodule diameter (mm)										
	Median (IQR)	23.0 (20.0–27.0)	18.0 (13.0–23.0)	< 0.001	24.0 (20.0–27.0)	20.0 (15.0–25.0)	< 0.001	23 (20.0–27.0)	17 (13.0–23.0)	< 0.001
Location*										
	LUL	177 (28.1%)	452 (71.9%)	0.002	79 (43.2%)	104 (56.8%)	0.227	98 (22.0%)	348 (78.0%)	0.040
	LLL	110 (35.7%)	198 (64.3%)		51 (46.4%)	59 (53.6%)		59 (29.8%)	139 (70.2%)	
	RUL	208 (26.3%)	584 (73.7%)		83 (44.4%)	104 (55.6%)		125 (20.7%)	480 (79.3%)	
	RML	52 (32.9%)	106 (67.1%)		20 (40.0%)	30 (60.0%)		32 (29.6%)	76 (70.4%)	
	RLL	134 (33.2%)	270 (66.8%)		64 (54.2%)	54 (45.8%)		70 (24.5%)	216 (75.5%)	
	NA	5 (71.4%)	2 (28.6%)		4 (80.0%)	1 (20.0%)		1 (50.0%)	1 (50.0%)	
Histologic findings										
	LUAD	504 (26.6%)	1388 (73.4%)	< 0.001	218 (43.3%)	286 (56.7%)	0.004	286 (20.6%)	1102 (79.4%)	< 0.001
	LUSC	59 (48.8%)	62 (51.2%)		27 (50.0%)	27 (50.0%)		32 (47.8%)	35 (52.2%)	
	SCLC	43 (84.3%)	8 (15.7%)		15 (83.3%)	3 (16.7%)		28 (84.8%)	5 (15.2%)	
	Other	80 (34.2%)	154 (65.8%)		41 (53.2%)	36 (46.8%)		39 (24.8%)	118 (75.2%)	
EGFR mutation										
	Yes	84 (34.0%)	163 (66.0%)	0.092	18 (60.0%)	12 (40.0%)	0.613	66 (30.4%)	151 (69.6%)	0.073
	No	116 (41.1%)	166 (58.9%)		26 (54.2%)	22 (45.8%)		90 (38.5%)	144 (61.5%)	
	NA	486 (27.5%)	1283 (72.5%)		257 (44.7%)	318 (55.3%)		229 (19.2%)	965 (80.8%)	
SUVmax										
	Median (IQR)	10.29 (7.05–13.65)	2.10 (0–5.50)	< 0.001	10.66 (7.30–13.88)	5.93 (2.91–10.35)	0.405	10.16 (6.57–13.43)	1.60 (0–3.81)	< 0.001
SUVmean										
	Median (IQR)	5.77 (3.87–7.91)	1.12 (0–2.95)	< 0.001	5.94 (4.07–8.09)	3.30 (1.59–6.01)	0.431	5.66 (3.56–7.74)	0.84 (0–1.99)	< 0.001
SULpeak										
	Median (IQR)	6.27 (4.18–8.58)	1.23 (0–3.04)	0.008	6.54 (4.45–8.75)	3.41 (1.69–6.21)	0.740	6.02 (3.98–8.24)	0.94 (0–2.15)	0.001
MTV										
	Median (IQR)	5.32 (3.38–7.83)	3.10 (0–6.00)	0.003	5.17 (3.20–7.90)	3.40 (1.96–5.88)	0.991	5.60 (3.46–7.83)	2.88 (0–6.10)	0.001
TLG										
	Median (IQR)	28.85 (16.83–48.59)	5.44 (0–13.53)	< 0.001	29.60 (17.55–50.13)	12.24 (5.11–24.63)	< 0.001	28.66 (16.05–47.68)	3.89 (0–10.64)	< 0.001

^*^LUL Left upper lobe, LLL Left lower lobe, RUL Right upper lobe, RML Right middle lobe, RLL Right lower lobe, LUAD lung adenocarcinoma, LUSC lung squamous cell carcinoma, SCLC small cell lung cancer, NA not available. EGFR, epidermal growth factor receptor; SUVmax, maximum standardized uptake value; SUVmean, mean standardized uptake value; SULpeak, standardized uptake values corrected for lean body mass; MTV, metabolic tumor volume; TLG, total lesion glycolysis

### Metastatic State

**Fig. 2:**
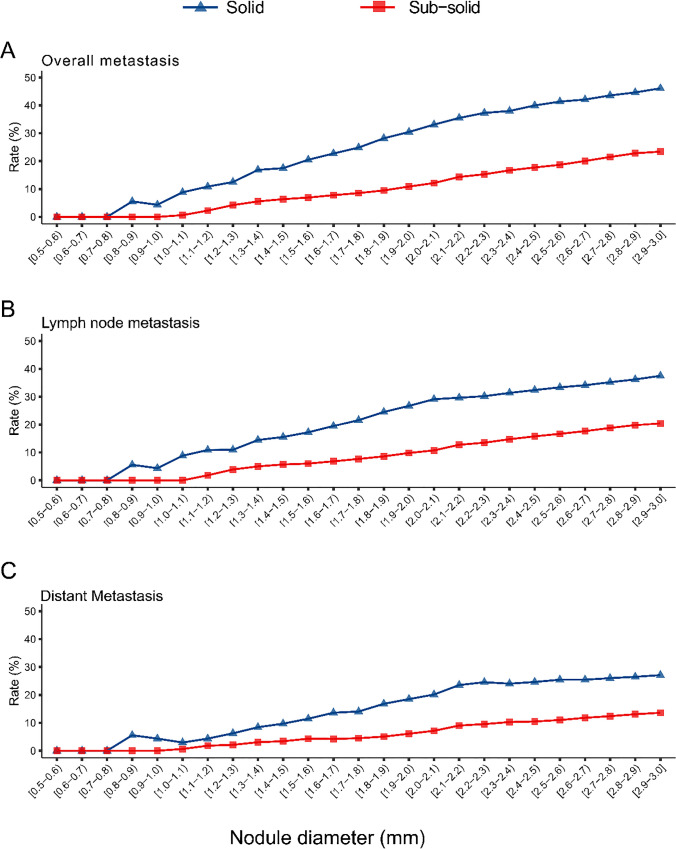
Comparison of Metastasis in Solid and Sub-solid Nodules in T1-Category Lung Cancer. Panels A-C depict nodule diameters (5–30 mm) on the X-axis versus detection rates of metastasis on the Y-axis: A represents overall metastasis, B indicates lymph node metastasis, and C shows distant metastasis. The detection rates of metastasis in solid nodules are marked in blue and in sub-solid nodules in red

No metastases (0%) were observed in patients with solid nodules < 8.0 mm and sub-solid nodules < 10.0 mm (Supplement Table 1). Metastases were found in 29.9% (686/2,298) of the patients, with a notably higher incidence among those with solid nodules (46.1%, 301/653) compared to those with sub-solid nodules (23.4%, 385/1,645). Furthermore, the overall detection rate of metastasis was consistently higher in solid nodules compared to sub-solid nodules within the same diameter range (Fig. 2A). As the diameter of both solid and sub-solid nodules increased, so did in the detection rates of metastasis. This trend was consistent across overall metastasis, lymph node metastasis, and distant metastasis for both nodules (Fig. 2B-C).

**Fig. 3:**
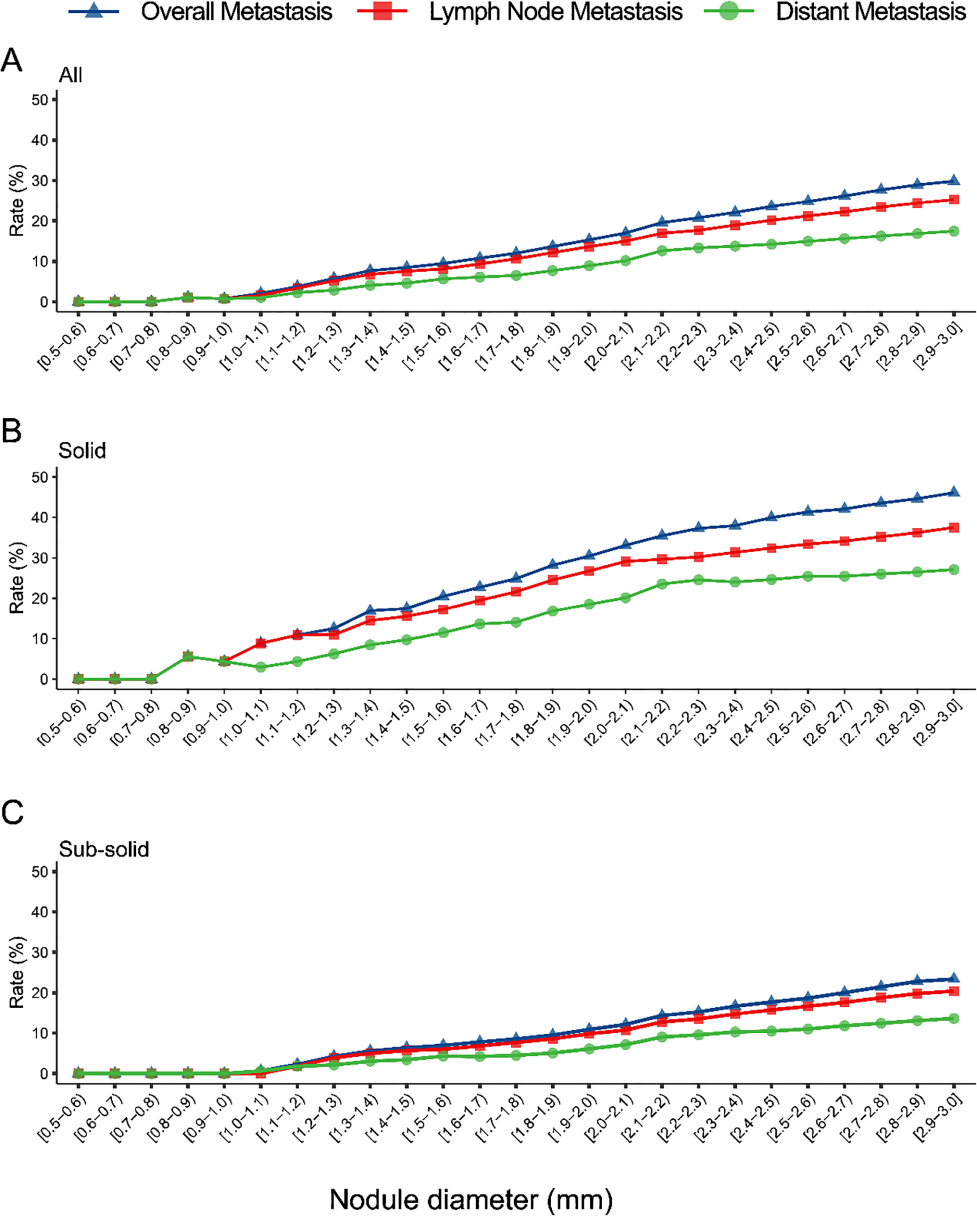
Detection Rates of Metastasis in T1-Category Lung Cancer with Solid and Sub-solid Nodules. Panels A-C depict nodule diameters (5–30 mm) on the X-axis versus detection rates of metastasis on the Y-axis: A represents all nodules, B indicates solid nodules, and C shows sub-solid nodules. The colors blue, red, and green represent the detection rates of overall metastasis, lymph node metastasis, and distant metastasis, respectively

Compared with distant metastasis, a higher detection rate of lymph node metastasis was observed within the same diameter of nodules (Fig. 3A and Supplement Table 1). This pattern persisted in both solid and sub-solid nodule groups (Fig. 3B-C). The detection rates of N1 and N2 lymph node metastases were comparable and exceeded that of N3 lymph node metastasis (Supplement Fig. 1A and Supplement Table 2). This trend remained consistent across solid and sub-solid nodules (Supplement Fig. 1B-C). Additionally, with increasing nodule diameter, the differences in distant metastasis rates (M1a-M1c) became more pronounced (Supplement Fig. 2A and Supplement Table 3), this trend was also observed in both solid and sub-solid nodules (Supplement Fig. 2B-C). In metastasis sites, ipsilateral hilar lymph nodes and bone were the most frequent sites for lymph node and distant metastases, respectively, with 300 cases of ipsilateral hilar lymph node metastasis and 217 cases of bone metastasis recorded. We also calculated the detection rates of metastasis diagnosed by PET/CT plus brain MRI separately, as shown in Supplement Fig. 3–7.

**Table 2:** The demographic and clinical-pathological features of T1-category lung cancer patients with post-operation

		All			Solid			Sub-solid		
		PET/CT plus MRI	CT only (or plus MRI)		PET/CT plus MRI	CT only (or plus MRI)		PET/CT plus MRI	CT only (or plus MRI)	
Gender				P value			P value			P value
	Male	744 (41.3%)	1059 (58.7%)	0.014	194 (52.3%)	177 (47.7%)	0.037	550 (38.4%)	882 (61.6%)	0.227
	Female	756 (37.4%)	1267 (62.6%)		121 (44.0%)	154 (56.0%)		635 (36.3%)	1113 (63.7%)	
Age (year)										
	Median (IQR)	59 (51–66)	57 (49–65)	< 0.001	62 (53–67)	60 (53–67)	0.496	59 (51–66)	56 (48–64)	< 0.001
Nodule diameter (mm)										
	Median (IQR)	18.0 (13.0–23.0)	14.0 (9.0–20.3)	< 0.001	20.0 (15.0–24.5)	17.0 (10.0–24.0)	< 0.001	17.0 (13.0–23.0)	14.0 (9.0–20.0)	< 0.001
Location										
	LUL	428 (41.8%)	597 (58.2%)	0.085	99 (60.4%)	65 (39.6%)	0.004	329 (38.2%)	532 (61.8%)	0.067
	LLL	174 (34.9%)	324 (65.1%)		51 (51.0%)	49 (49.0%)		123 (30.9%)	275 (69.1%)	
	RUL	548 (39.8%)	829 (60.2%)		92 (46.0%)	108 (54.0%)		456 (38.7%)	721 (61.3%)	
	RML	99 (35.9%)	177 (64.1%)		27 (37.5%)	45 (62.5%)		72 (35.3%)	132 (64.7%)	
	RLL	251 (38.6%)	399 (61.4%)		46 (41.8%)	64 (58.2%)		205 (38.0%)	335 (62.0%)	
Pathologic risk factors										
Pleural invasion										
	Yes	192 (42.6%)	259 (57.4%)	0.119	81 (56.6%)	62 (43.4%)	0.033	111 (36.0%)	197 (64.0%)	0.640
	No	1308 (38.8%)	2067 (61.2%)		234 (46.5%)	269 (53.5%)		1074 (37.4%)	1798 (62.6%)	
Vascular cancer embolus										
	Yes	174 (51.9%)	161 (48.1%)	< 0.001	90 (62.5%)	54 (37.5%)	< 0.001	84 (44.0%)	107 (56.0%)	0.048
	No	1326 (38.0%)	2165 (62.0%)		225 (44.8%)	277 (55.2%)		1101 (36.8%)	1888 (63.2%)	
Spread through air spaces										
	Yes	120 (94.5%)	7 (5.5%)	< 0.001	68 (95.8%)	3 (4.2%)	< 0.001	52 (92.9%)	4 (7.1%)	< 0.001
	No	1380 (37.3%)	2139 (62.7%)		247 (43.0%)	328 (57.0%)		1133 (36.3%)	1991 (63.7%)	
Nerve invasion										
	Yes	18 (75.0%)	6 (25.0%)	< 0.001	11 (91.7%)	1 (8.3%)	0.003	7 (58.3%)	5 (41.7%)	0.144
	No	1482 (39.0%)	2320 (61.0%)		304 (47.9%)	330 (52.1%)		1178 (37.2%)	1990 (62.8%)	
Bronchial invasion										
	Yes	23 (39.0%)	36 (61.0%)	0.972	9 (50.0%)	9 (50.0%)	0.915	14 (34.1%)	27 (65.9%)	0.678
	No	1477 (39.2%)	2290 (60.8%)		306 (48.7%)	322 (51.3%)		1171 (37.3%)	1968 (62.7%)	
Vascular invasion										
	Yes	6 (42.9%)	8 (57.1%)	0.779	3 (75.0%)	1 (25.0%)	0.362	3 (30.0%)	7 (70.0%)	0.753
	No	1494 (39.2%)	2318 (60.8%)		312 (48.6%)	330 (51.4%)		1182 (37.3%)	1988 (62.7%)	
Histologic findings										
	LUAD	1309 (37.4%)	2187 (62.6%)	< 0.001	257 (47.5%)	284 (52.5%)	0.003	1052 (35.6%)	1903 (64.4%)	< 0.001
	LUSC	44 (34.4%)	84 (65.6%)		22 (40.0%)	33 (60.0%)		22 (30.1%)	51 (69.9%)	
	SCLC	5 (26.3%)	14 (73.7%)		3 (50.0%)	3 (50.0%)		2 (15.4%)	11 (84.6%)	
	Other	142 (77.6%)	41 (22.4%)		33 (75.0%)	11 (25.0%)		109 (78.4%)	30 (21.6%)	
Invasiveness										
	AAH, AIS	34 (14.4%)	202 (85.6%)	< 0.001	4 (18.2%)	18 (81.8%)	0.007	30 (14.0%)	184 (86.0%)	< 0.001
	MIA	193 (25.3%)	571 (74.7%)		17 (38.6%)	27 (61.4%)		176 (24.4%)	544 (75.6%)	
	IA	1082 (43.3%)	1414 (56.7%)		236 (49.7%)	239 (50.3%)		846 (41.9%)	1175 (58.1%)	
IA combined with high-grade patterns*										
	Yes	459 (50.1%)	458 (49.9%)	< 0.001	165 (60.7%)	107 (39.3%)	< 0.001	294 (45.6%)	351 (54.4%)	< 0.001
	No	850 (33.0%)	1729 (67.0%)		92 (34.2%)	177 (65.8%)		758 (32.8%)	1552 (67.2%)	
EGFR mutation										
	Yes	151 (19.7%)	617 (80.3%)	0.592	8 (9.0%)	81 (91.0%)	0.034	143 (21.1%)	536 (78.9%)	0.958
	No	150 (20.8%)	572 (79.2%)		21 (19.8%)	85 (80.2%)		129 (20.9%)	487 (79.1%)	
	NA	1199 (51.3%)	1137 (48.7%)		286 (63.4%)	165 (36.6%)		913 (48.4%)	972 (51.6%)	
SUVmax										
	Median (IQR)	2.04 (0–5.05)			6.00 (2.86–10.35)			1.55 (0–3.55)		
SUVmean										
	Median (IQR)	1.07 (0–2.73)			3.41 (1.55–6.00)			0.83 (0–1.92)		
SULpeak										
	Median (IQR)	1.18 (0–2.84)			3.42 (1.69–6.20)			0.92 (0–2.05)		
MTV										
	Median (IQR)	3.09 (0–5.94)			3.38 (1.94–5.62)			2.80 (0–6.01)		
TLG										
	Median (IQR)	5.13 (0–12.80)			11.62 (5.18–23.78)			3.73 (0–10.13)		

LUL, Left upper lobe; LLL, Left lower lobe; RUL, Right upper lobe; RML, Right middle lobe; RLL, Right lower lobe; LUAD, lung adenocarcinoma; LUSC, lung squamous cell carcinoma; SCLC, small cell lung cancer; AAH, Atypical adenomatous hyperplasia; AIS, Adenocarcinoma in situ; MIA, Minimally invasive adenocarcinoma; IA, Invasive adenocarcinoma; NA not available. *, High-grade patterns include solid, micropapillary, and complex glandular patterns. EGFR, epidermal growth factor receptor; SUVmax, maximum standardized uptake value; SUVmean, mean standardized uptake value; SULpeak, standardized uptake values corrected for lean body mass; MTV, metabolic tumor volume; TLG, total lesion glycolysis

**Table 3:** Univariate and multivariate analysis of overall survival for T1-category lung cancer patients

			Univariate Analysis			Multivariate Analysis		
Group			HR	95% CI	P value	HR	95% CI	P value
	PET/CT plus MRI	Ref						
	CT only (or plus MRI)		1.90	1.16–3.14	0.011	2.64	1.58–4.40	< 0.001
Gender								
	Male	Ref						
	Female		0.53	0.36–0.77	0.001	0.76	0.51–1.14	0.184
Pleural invasion								
	No	Ref						
	Yes		3.85	2.61–5.66	< 0.001	2.40	1.57–3.67	< 0.001
Vascular cancer embolus								
	No	Ref						
	Yes		7.07	4.81–10.39	< 0.001	4.41	2.87–6.75	< 0.001
Spread through air spaces								
	No	Ref						
	Yes		0.54	0.07–3.88	0.539	0.29	0.04–2.14	0.223
Nerve invasion								
	No	Ref						
	Yes		3.83	0.94–15.51	0.060	0.96	0.23–4.07	0.956
Bronchial invasion								
	No	Ref						
	Yes		2.19	0.81–5.94	0.124	0.90	0.32–2.52	0.834
Vascular invasion								
	No	Ref						
	Yes		5.78	1.43–23.42	0.014	1.87	0.45–7.77	0.389
Location								
	LUL	Ref						
	LLL		1.39	0.80–2.43	0.243	1.31	0.74–2.32	0.349
	RUL		0.83	0.51–1.36	0.460	1.00	0.61–1.64	0.989
	RML		1.43	0.73–2.79	0.295	1.34	0.68–2.65	0.404
	RLL		0.91	0.51–1.63	0.748	1.02	0.57–1.83	0.948
Histologic findings								
	LUAD	Ref						
	LUSC		2.98	1.59–5.56	< 0.001	1.94	0.99–3.80	0.053
	SCLC		1.87	0.26–13.38	0.535	1.11	0.15–8.32	0.918
	Other		1.54	0.78–3.05	0.215	1.79	0.89–3.61	0.103
Component								
	Solid	Ref						
	Sub-solid		0.42	0.28–0.63	< 0.001	0.79	0.51–1.22	0.285
Diameter								
	Solid < 8 mm & Sub-solid < 10 mm	Ref						
	Solid ≥ 8 mm & Sub-solid ≥ 10 mm		7.37	2.72–20.00	< 0.001	4.49	1.63–12.40	0.004
Age (year)								
	< 55	Ref						
	≥ 55		2.23	1.44–3.45	< 0.001	1.62	1.04–2.52	0.035

HR, hazard ratio; CI, confidence interval; LUL, Left upper lobe; LLL, Left lower lobe; RUL, Right upper lobe; RML, Right middle lobe; RLL, Right lower lobe; LUAD, lung adenocarcinoma; LUSC, lung squamous cell carcinoma; SCLC, small cell lung cancer

To confirm the cut-off values of metabolic parameters on PET/CT for the 0% detection rate of metastasis, patients with solid nodules < 8.0 mm and sub-solid nodules < 10.0 mm were excluded (no metastasis was found below these thresholds) and then calculated the AUC of maximum specificity in each metabolic parameter (SUVmax/SUVmean/SULpeak/MTV/TLG). The established cut-off for discriminating metastasis on PET/CT metabolic parameters were as follows: 1.09 (SUVmax), 0.26 (SUVmean), 0.31 (SULpeak), 0.55 (MTV), and 0.81 (TLG), respectively (Supplement Fig. 8).

### Associations of PET/CT plus MRI with survival

**Fig. 4:**
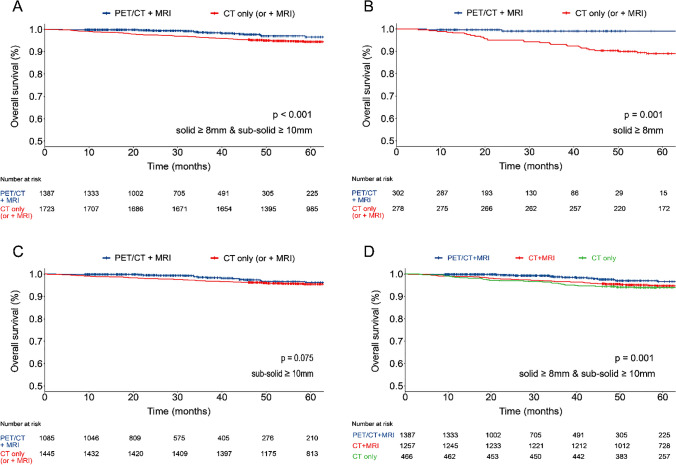
Overall Survival in T1-Category Lung Cancer Patients with Solid (≥ 8 mm) and Sub-Solid (≥ 10 mm) Nodules, Analyzed by PET/CT plus MRI, CT plus MRI, and CT only. Panel A-D displays the patient’s overall survival time (in months) on the X-axis, with the Y-axis showing the overall survival rate of post-surgical tumor resection. A: solid nodule ≥ 8 mm & sub-solid nodule ≥ 10 mm; B: solid nodule ≥ 8 mm; C: sub-solid nodule ≥ 10 mm and D: solid nodule ≥ 8 mm & sub-solid nodule ≥ 10 mm among PET/CT plus MRI, CT plus MRI, and CT only. The overall survival was estimated using the Kaplan–Meier (K-M) methodology, and the between-group difference in overall survival was assessed by stratified log-rank test. Time for patients who were alive or lost to follow-up were censored at the time of last contact: April 20, 2023. Blue and red indicate overall survival for patients undergoing PET/CT plus MRI and CT only (or plus MRI) in panels A-C, respectively. Blue, red and green indicate overall survival for patients undergoing PET/CT plus MRI, CT plus MRI, and CT only in panel D. The column below (No. at risk) lists the number of patients

**Fig. 5:**
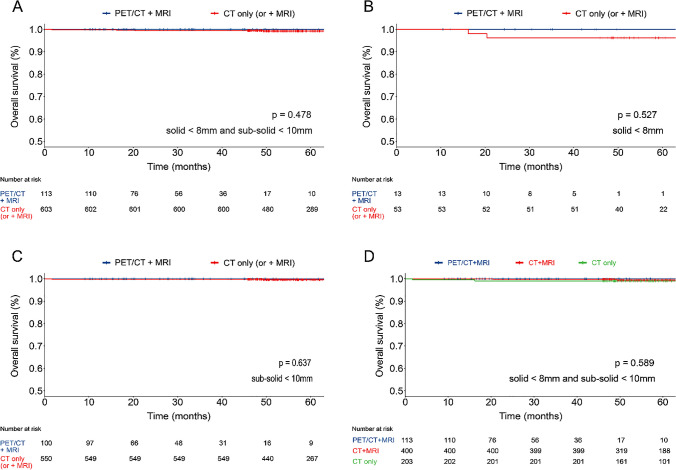
Overall Survival in T1-Category Lung Cancer Patients with Solid (< 8 mm) and Sub-Solid (< 10 mm) Nodules, Analyzed by PET/CT plus MRI, CT plus MRI, and CT only. Panel A-D displays the patient’s overall survival time (in months) on the X-axis, with the Y-axis showing the overall survival rate of post-surgical tumor resection. A: solid nodule < 8 mm & sub-solid nodule < 10 mm; B: solid nodule < 8 mm; C: sub-solid nodule < 10 mm and D: solid nodule < 8 mm & sub-solid nodule < 10 mm among PET/CT plus MRI, CT plus MRI, and CT only. The overall survival was estimated using the Kaplan–Meier (K-M) methodology, and the between-group difference in overall survival was assessed by a stratified log-rank test. Time for patients who were alive or lost to follow-up were censored at the time of last contact: April 20, 2023. Blue and red indicate overall survival for patients undergoing PET/CT plus MRI and CT only (or plus MRI) in panels A-C, respectively. Blue, red and green indicate overall survival for patients undergoing PET/CT plus MRI, CT plus MRI, and CT only in panel D. The column below (No. at risk) lists the number of patients

For the survival analysis, a cohort of 4,264 patients underwent surgery was assembled, comprising 1,683 individuals who underwent PET/CT plus MRI and 2,581 who underwent either CT plus MRI or CT only. Out of 2,581 patients, 1,792 patients underwent contrast-enhanced CT and CT scans simultaneously (1,792/2,581, 69.4%). Patients identified metastasis (183 from the PET/CT plus MRI group and 255 from the CT only or plus MRI group) were excluded. The remaining 3,826 patients, consisting of 744 males (41.3%) in the PET/CT plus MRI group (p = 0.014), which was characterized by an elder age profile (median age, 59 years; IQR, 51–66 years; p < 0.001), larger nodule diameter (median diameter, 18.0 mm; IQR, 13.0–23.0 mm; p < 0.001), more other cancer type, and invasive adenocarcinomas (p < 0.001 for both). Nodule locations were comparable between two groups (p = 0.085) (Table 2). Among the risk factors for postoperative pathological, the PET/CT plus MRI group exhibited higher incidences of vascular cancer thrombus (p < 0.001), spread through air spaces (STAS; p < 0.001), and nerve invasion (p < 0.001). No differences were observed in pleural invasion (p = 0.119), bronchial invasion (p = 0.972), and vascular invasion (p = 0.779). EGFR mutation testing was detected in 1,490 (38.9%) patients, with 768 positive (p = 0.592). Data regarding solid and sub-solid nodules are detailed in Table 2. Notably, patients who underwent PET/CT plus MRI demonstrated a longer OS compared to those who received CT only (or plus MRI) in solid nodules ≥ 8.0 mm & sub-solid nodules ≥ 10.0 mm (HR, 0.44; 95%CI, 0.27–0.72; p < 0.001); in solid nodules ≥ 8.0 mm (HR, 0.12; 95%CI, 0.03–0.49; p < 0.001); and in sub-solid nodules ≥ 10.0 mm (HR, 0.61; 95%CI, 0.35–1.05; p = 0.075) (Fig. 4A-C). No difference in OS was observed in solid nodules < 8.0 mm & sub-solid nodules < 10.0 mm (p = 0.478); in solid nodules < 8.0 mm (p = 0.527); and in sub-solid nodules < 10.0 mm (p = 0.637), as depicted in Fig. 5A-C. In addition, several potential clinical factors that might impact the OS were examined using the univariate and multivariate Cox regression analysis, indicating that without PET/CT plus MRI examination (HR, 2.64; 95%CI, 1.58–4.40; p < 0.001), pleural invasion (HR, 2.40; 95%CI, 1.57–3.67; p < 0.001), vascular cancer embolus (HR, 4.41; 95%CI, 2.87–6.75; p < 0.001), solid ≥ 8 mm & sub-solid ≥ 10 mm (HR, 4.49; 95%CI, 1.63–12.40; p = 0.004) and age ≥ 55 years (HR, 1.62; 95%CI, 1.04–2.52; p = 0.035) were associated with the poor prognosis (Table 3).

When the cohort undergoing CT only (or plus MRI) was subdivided into those receiving CT plus MRI and those receiving CT only, difference in OS was observed among groups in solid nodules ≥ 8.0 mm and sub-solid nodules ≥ 10.0 mm (PET/CT plus MRI versus CT plus MRI versus CT only, p = 0.001) (Fig. 4D), and no difference in OS was observed in solid nodules < 8.0 mm and sub-solid nodules < 10.0 mm (p = 0.923) (Fig. 5D). After the PSM procedure, all baseline variates were balanced (p > 0.05), and the demographic and clinical-pathological features can be found in Supplement Table 4. The differences in OS of those who underwent PET/CT plus MRI after PSM were consistent with that before PSM (Supplement Fig. 9–10). In clinical T1-category lung cancer patients who underwent PET/CT plus MRI, metabolic parameters could be the predictors for poor prognosis, compared with metabolic parameters lower than cut-off value, patients with higher than cut-off had shorter OS: SUVmax (HR, 12.94; 95%CI, 2.98–56.09; p < 0.001), SUVmean (HR, 11.33; 95%CI, 2.61–49.13; p < 0.001), SULpeak (HR, 9.65; 95%CI, 3.47–26.83; p < 0.001), MTV (HR, 9.16, 95%CI, 1.22–68.73; p = 0.031), and TLG (HR, 12.06; 95%CI, 2.78–53.25; p < 0.001)(Supplement Fig. 11–15).

Histologically, lung adenocarcinoma predominated, accounting for 82.3% (1,892/2,298) of the cases. The detection rate of metastasis in lung adenocarcinoma can be found in Supplement Table 5–7, while the detection rate of metastasis in lung non-adenocarcinoma were recorded in Supplement Table 8–10. Correspondingly, the results compared with lung non-adenocarcinoma were shown in Supplement Fig. 16–19. Lung adenocarcinoma accounted for 91.4% (3,496/3,826) of the cases included in the prognosis analysis and was further classified into atypical adenomatous hyperplasia (AAH), adenocarcinoma in situ (AIS), minimally invasive adenocarcinoma (MIA), and invasive adenocarcinoma (IA). Significant survival differences were observed among these groups (p < 0.001) (Supplement Fig. 20A). Furthermore, no significant differences in survival rates were detected among the AAH, AIS, and MIA subtypes when assessed using PET/CT combined with MRI (p = 0.126) (Supplement Fig. 20B), significant survival differences were found in IA with or without high-grade patterns (p = 0.014 and p = 0.013, respectively) (Supplement Fig. 20C-D). EGFR mutation testing was performed in 1490 patients, and the detection rates of metastasis for EGFR mutant and EGFR wild-type patients was detailed in Supplement Table 11–16.

## Discussion

PET/CT and brain MRI are widely recognized as effective methods for detecting metastases and staging of lung cancer, offering significant advantages over contrast-enhanced CT and other radiological techniques [16, 17]. PET/CT could intricately delineate viable tumor tissue and metastasis by analyzing tumor metabolism, provide clinically precise lymph node staging [6], and avert unnecessary treatments intended for cure [18]. Complementarily, MRI serves as an efficacious adjunctive tool, particularly adept at detecting brain metastases in lung cancer patients [17]. In our study, we have stratified metastasis risk in clinical T1-category lung cancer patients by lesion type and diameter, enabling us to ascertain critical diameter thresholds for detecting metastases using PET/CT plus MRI, thereby seeking to refine existing clinical guidelines.

This study reveals a direct correlation between the increased detection rate of metastasis and the diameter for both solid and sub-solid nodules. Notably, solid nodules presented a heightened risk at comparable diameters. Additionally, we observed similar detection rates for lymph node involvement at N1 and N2 stations, which were significantly higher than those at N3 stations within the same diameter range. The incidence of M1a metastasis outstripped that of M1b and M1c metastases, aligning with outcomes reported in previous research [2, 12, 13, 19]. Demographically, our findings indicate that lung cancer patients with metastasis are predominantly male and tend to have larger nodule diameters, and variations in detection rates of metastasis may be related to the locations of the nodules and their histologic findings.

Our research delineates clear thresholds for metastasis detection based on lesions' diameters and nodules types: solid nodules measuring 8 mm and sub-solid nodules measuring 10 mm exhibited detection rates of metastasis of 0%. Further, no significant differences in OS were observed among patients with solid nodules < 8.0 mm and sub-solid nodules < 10.0 mm. Therefore, patients with smaller nodules demonstrated a lower propensity for metastasis, suggesting that PET/CT plus MRI might be superfluous in their cases. These examinations were still essential for clinical T1-category lung cancer patients with solid nodules ≥ 8.0 mm and sub-solid nodules ≥ 10.0 mm; for these patients, CT alone or plus MRI was insufficient for detecting the potential metastases after surgery and might led to poor prognosis.

Previous research indicated that metabolic parameters could be prognostic predictors [20–23]. Moon et al. found that SUVmax is an independent prognostic indicator capable of significantly improving risk stratification, Kurtipek et al. indicated that SUVmean and MTV had a significant impact on prognosis [21], Ling et al. and Lee et al. pointed out that MTV and TLG may have predictive value [22, 23]. The findings of this study confirm that these metabolic parameters can be effectively used as prognostic predictors for T1-category lung cancer.

In this study, we cautiously propose that PET/CT and MRI examinations may not be necessary for clinical T1-category lung cancer patients with solid nodules < 8.0 mm and sub-solid nodules < 10.0 mm. This recommendation is based on the 0% detection rate of metastasis in these groups and lack of significant differences in OS between patients with and without PET/CT plus MRI examinations. Furthermore, our findings suggest that lower detection rate of metastasis in sub-solid nodules in comparable diameter, possibly attributable to their lower metastatic capability compared to solid nodules. This is supported by Ye et al., who reported a lower prevalence of lymph node metastasis in patients with part-solid nodules (2.2%) compared to those with pure solid nodules (27%) [24]. Similarly, Choi et al. observed a significant difference in the prevalence of lymph node metastasis between pure-solid nodules and mixed ground-glass nodules [25], with additional studies corroborating these findings [26–28]. GGO-predominant lung cancers are generally characterized by less active metabolism and immune microenvironment than solid nodules, which may underlie their more indolent clinical course and less aggressive invasiveness [29]. Moreover, the variance in gene mutations (e.g., TP53) between solid and sub-solid lesions may contribute to their differing metastatic potentials [29, 30]. Upon examining baseline data for prognostic analysis, it was observed that the cohort undergoing PET/CT plus MRI, typically of advanced age, presented larger and more aggressive nodular characteristics. Notwithstanding these factors, this group demonstrated an unexpectedly superior prognosis, even after the matching variates were propensity-score matched. This may be attributed to the enhanced diagnostic accuracy of PET/CT combined with MRI in excluding metastasis, thereby informing, and potentially altering subsequent therapeutic strategies.

To the best of our knowledge, this study represents the inaugural effort to represent the detection rate of metastasis of PET/CT plus MRI in clinical T1-category lung cancer, specifically addressing the variations across solid and sub-solid nodules of different diameters. While the NCCN guidelines advocate for PET/CT in all lung cancer cases, there remains a lack of relative data. Meanwhile, MRI is recommended for all NSCLC patients except for those with peripheral stage IA NSCLC [6, 7]. The use of brain MRI in early-stage lung cancer is controversial, about a quarter of patients with stage IA NSCLC receive brain imaging at diagnosis, despite national recommendations against the practice [31]. Nam et al. [32] found that no difference was observed between patients with clinical stage IA NSCLC who underwent brain MRI and those who did not in terms of OS. However, relevant studies did not stratify the metastatic risk of lung cancer based on lesions' size and morphological characteristics simultaneously, therefore, for clinical T1-category lung cancer patients, the conclusions may be incomplete and imprecise. Our study stratifies patients based on metastatic risk in terms of the lesions' size and morphological characteristics in clinical T1-category lung cancer patients. There was a difference in OS between clinical T1-category lung cancer patients with solid nodules ≥ 8.0 mm and sub-solid nodules ≥ 10.0 mm who underwent brain MRI and those who did not, but the difference was not statistically significant. Significantly, our study suggests an economic advantage for clinical T1-category lung cancer patients with smaller nodules (solid nodules <8 mm and sub-solid nodules <10 mm), proposing that they could be exempted from PET/CT plus MRI examinations. This recommendation could reduce unnecessary medical interventions, thereby saving costs and facilitating the healthcare resources allocation [33].

It is crucial to consider the local medical and economic circumstances when deciding whether to utilize or forgo combined PET/CT and MRI. Additionally, although the sensitivity of PET/CT in lung cancer is generally recognized, 2D mode image acquisitions used in this study is below the state of the art, besides, the specificity is limited [34]. More accurate methods/tools for metastasis detection, like molecular biomarkers and artificial intelligence (AI)-based diagnosis, particularly in clinical T1-category lung cancer patients [17, 35–38] This study presents several limitations. Firstly, the nature of retrospective studies inherently introduces potential biases in patient selection and evaluation. These biases may stem from factors, including the non-randomized selection of participants and reliance on existing records. Our findings necessitate further validation through a prospective multi-center study. Secondly, the genetic profiling in our study was predominantly focused on EGFR mutations, with only a subset of patients being assessed for other driver gene mutations. Thirdly, all the PET scanning was performed by using 2D mode, which is below the state of the art and may reduce sensitivity. Image acquisition using 3D mode is warrant to validified our findings.

In conclusion, our findings indicate that PET/CT plus MRI may not be necessary for clinical T1-category lung cancer patients with solid nodules < 8.0 mm and sub-solid nodules < 10.0 mm, considering their lower metastatic potency. Conversely, these imaging modalities are recommended for patients with solid nodules ≥ 8.0 mm and sub-solid nodules ≥ 10.0 mm, where the risk of metastasis is comparatively higher.

### Supplementary Information

Below is the link to the electronic supplementary material.

Supplementary file1 (DOCX 128 KB)

## Data Availability

This study was a retrospective single-center analysis, and the current study are available from the corresponding author on reasonable request.
