# Supplementary material for: The impact of PET/CT and brain MRI for metastasis detection among patients with clinical T1-category lung cancer: Findings from a large-scale cohort study
Source: Eur J Nucl Med Mol Imaging. 2024 May 9;51(11):3400–16. doi: 10.1007/s00259-024-06740-8 (PMC11369054; doi:10.1007/s00259-024-06740-8)
Supplement: Supplementary file 1 — Supplementary file1 (DOCX 128 KB) [file 259_2024_6740_MOESM1_ESM.docx]

**Legend**

**Supplement Table 1: The detection rates of metastasis in T1-category lung cancer patients (overall metastasis, lymph node metastasis and distant metastasis).**

**Supplement Table 2: The detection rates of lymph node metastasis in T1-category lung cancer patients (N1-N3).**

**Supplement Table 3: The detection rates of distant metastasis in T1-category lung cancer patients (M1a-M1c).**

**Supplement Table 4: The demographic and clinical-pathological features of T1-category lung cancer patients with pulmonary nodules after propensity score matching.**

**Supplement Table 5: The detection rates of metastasis in T1-category lung adenocarcinoma patients (overall metastasis, lymph node metastasis and distant metastasis).**

**Supplement Table 6: The detection rates of lymph node metastasis in T1-category lung adenocarcinoma patients (N1-N3).**

**Supplement Table 7: The detection rates of distant metastasis in T1-category lung adenocarcinoma patients (M1a-M1c).**

**Supplement Table 8:** **The detection rates of metastasis in T1-category non-adenocarcinoma lung cancer patients (overall metastasis, lymph node metastasis and distant metastasis).**

**Supplement Table 9: The detection rates of lymph node metastasis in T1-category non-adenocarcinoma lung cancer patients (N1-N3).**

**Supplement Table 10: The detection rates of distant metastasis in T1-category non-adenocarcinoma lung cancer patients (M1a-M1c).**

**Supplement Table 11: The detection rates of metastasis in T1-category lung cancer patients with EGFR mutation (overall metastasis, lymph node metastasis and distant metastasis).**

**Supplement Table 12: The detection rates of lymph node metastasis in T1-category lung cancer patients with EGFR mutation (N1-N3).**

**Supplement Table 13: The detection rates of distant metastasis in T1-category lung cancer patients with EGFR mutation (M1a-M1c).**

**Supplement Table 14: The detection rates of metastasis in T1-category lung cancer patients with EGFR wild-type (overall metastasis, lymph node metastasis and distant metastasis).**

**Supplement Table 15: The detection rates of lymph node metastasis in T1-category lung cancer patients with EGFR wild-type (N1-N3).**

**Supplement Table 16: The detection rates of distant metastasis in T1-category lung cancer patients with EGFR wild-type (M1a-M1c).**

| **Supplement Table 1: The detection rates of metastasis in T1-category lung cancer patients (overall metastasis, lymph node metastasis and distant metastasis).** | | | | | | | | | | | | | | | | | | | |
| --- | --- | --- | --- | --- | --- | --- | --- | --- | --- | --- | --- | --- | --- | --- | --- | --- | --- | --- | --- |
|  | | **All** | | |  | | |  | | **Solid** | |  | | |  | | | **Sub-solid** | |
| Diameter (mm) | Overall  metastasis | | Lymph node  metastasis | Distant  metastasis | |  |  | | Overall  metastasis | Lymph node  metastasis | Distant  metastasis | |  |  | | Overall  metastasis | Lymph node  metastasis | | Distant  metastasis |
| [5.0-6.0) | 0.0% | | 0.0% | 0.0% | |  | [5.0-6.0) | | 0.00% | 0.00% | 0.00% | |  | [5.0-6.0) | | 0.0% | 0.0% | | 0.0% |
| [6.0-7.0) | 0.0% | | 0.0% | 0.0% | |  | [6.0-7.0) | | 0.00% | 0.00% | 0.00% | |  | [6.0-7.0) | | 0.0% | 0.0% | | 0.0% |
| [7.0-8.0) | 0.0% | | 0.0% | 0.0% | |  | [7.0-8.0) | | 0.00% | 0.00% | 0.00% | |  | [7.0-8.0) | | 0.0% | 0.0% | | 0.0% |
| [8.0-9.0) | 1.1% | | 1.1% | 1.1% | |  | [8.0-9.0) | | 5.56% | 5.56% | 5.56% | |  | [8.0-9.0) | | 0.0% | 0.0% | | 0.0% |
| [9.0-10.0) | 0.7% | | 0.7% | 0.7% | |  | [9.0-10.0) | | 4.35% | 4.35% | 4.35% | |  | [9.0-10.0) | | 0.0% | 0.0% | | 0.0% |
| [10.0-11.0) | 2.1% | | 1.6% | 1.0% | |  | [10.0-11.0) | | 8.82% | 8.82% | 2.94% | |  | [10.0-11.0) | | 0.6% | 0.0% | | 0.6% |
| [11.0-12.0) | 3.7% | | 3.4% | 2.2% | |  | [11.0-12.0) | | 10.87% | 10.87% | 4.35% | |  | [11.0-12.0) | | 2.3% | 1.8% | | 1.8% |
| [12.0-13.0) | 5.8% | | 5.2% | 2.9% | |  | [12.0-13.0) | | 12.50% | 10.94% | 6.25% | |  | [12.0-13.0) | | 4.2% | 3.9% | | 2.1% |
| [13.0-14.0) | 7.7% | | 6.8% | 4.1% | |  | [13.0-14.0) | | 16.87% | 14.46% | 8.43% | |  | [13.0-14.0) | | 5.6% | 5.0% | | 3.1% |
| [14.0-15.0) | 8.5% | | 7.6% | 4.6% | |  | [14.0-15.0) | | 17.48% | 15.53% | 9.71% | |  | [14.0-15.0) | | 6.4% | 5.7% | | 3.4% |
| [15.0-16.0) | 9.5% | | 8.1% | 5.6% | |  | [15.0-16.0) | | 20.49% | 17.21% | 11.48% | |  | [15.0-16.0) | | 6.9% | 6.0% | | 4.3% |
| [16.0-17.0) | 10.8% | | 9.4% | 6.1% | |  | [16.0-17.0) | | 22.73% | 19.48% | 13.64% | |  | [16.0-17.0) | | 7.8% | 6.8% | | 4.2% |
| [17.0-18.0) | 12.0% | | 10.6% | 6.5% | |  | [17.0-18.0) | | 24.86% | 21.62% | 14.05% | |  | [17.0-18.0) | | 8.5% | 7.7% | | 4.5% |
| [18.0-19.0) | 13.7% | | 12.1% | 7.7% | |  | [18.0-19.0) | | 28.18% | 24.55% | 16.82% | |  | [18.0-19.0) | | 9.5% | 8.6% | | 5.1% |
| [19.0-20.0) | 15.3% | | 13.6% | 8.9% | |  | [19.0-20.0) | | 30.45% | 26.75% | 18.52% | |  | [19.0-20.0) | | 10.9% | 9.8% | | 6.1% |
| [20.0-21.0) | 17.0% | | 15.0% | 10.2% | |  | [20.0-21.0) | | 33.09% | 29.14% | 20.14% | |  | [20.0-21.0) | | 12.1% | 10.7% | | 7.2% |
| [21.0-22.0) | 19.6% | | 16.9% | 12.6% | |  | [21.0-22.0) | | 35.47% | 29.66% | 23.55% | |  | [21.0-22.0) | | 14.3% | 12.7% | | 9.0% |
| [22.0-23.0) | 20.8% | | 17.7% | 13.3% | |  | [22.0-23.0) | | 37.29% | 30.23% | 24.58% | |  | [22.0-23.0) | | 15.3% | 13.5% | | 9.6% |
| [23.0-24.0) | 22.1% | | 18.9% | 13.8% | |  | [23.0-24.0) | | 37.97% | 31.39% | 24.05% | |  | [23.0-24.0) | | 16.7% | 14.7% | | 10.3% |
| [24.0-25.0) | 23.6% | | 20.1% | 14.2% | |  | [24.0-25.0) | | 39.95% | 32.42% | 24.66% | |  | [24.0-25.0) | | 17.7% | 15.8% | | 10.5% |
| [25.0-26.0) | 24.8% | | 21.2% | 14.9% | |  | [25.0-26.0) | | 41.34% | 33.40% | 25.47% | |  | [25.0-26.0) | | 18.7% | 16.7% | | 11.0% |
| [26.0-27.0) | 26.2% | | 22.2% | 15.6% | |  | [26.0-27.0) | | 42.08% | 34.15% | 25.47% | |  | [26.0-27.0) | | 20.0% | 17.6% | | 11.8% |
| [27.0-28.0) | 27.7% | | 23.4% | 16.2% | |  | [27.0-28.0) | | 43.54% | 35.22% | 26.02% | |  | [27.0-28.0) | | 21.5% | 18.8% | | 12.4% |
| [28.0-29.0) | 28.9% | | 24.4% | 16.8% | |  | [28.0-29.0) | | 44.63% | 36.24% | 26.51% | |  | [28.0-29.0) | | 22.8% | 19.8% | | 13.1% |
| [29.0-30.0] | 29.9% | | 25.3% | 17.4% | |  | [29.0-30.0] | | 46.09% | 37.52% | 27.11% | |  | [29.0-30.0] | | 23.4% | 20.4% | | 13.6% |

| **Supplement Table 2: The detection rates of lymph node metastasis in T1-category lung cancer patients (N1-N3).** | | | | | | | | | | | | |  |
| --- | --- | --- | --- | --- | --- | --- | --- | --- | --- | --- | --- | --- | --- |
|  | **All** | | |  |  | **Solid** | | |  |  | **Sub-solid** | | |
| Diameter (mm) | N1 | N2 | N3 |  |  | N1 | N2 | N3 |  |  | N1 | N2 | N3 |
| [5.0-6.0) | 0.0% | 0.0% | 0.0% |  | [5.0-6.0) | 0.00% | 0.00% | 0.00% |  | [5.0-6.0) | 0.0% | 0.0% | 0.0% |
| [6.0-7.0) | 0.0% | 0.0% | 0.0% |  | [6.0-7.0) | 0.00% | 0.00% | 0.00% |  | [6.0-7.0) | 0.0% | 0.0% | 0.0% |
| [7.0-8.0) | 0.0% | 0.0% | 0.0% |  | [7.0-8.0) | 0.00% | 0.00% | 0.00% |  | [7.0-8.0) | 0.0% | 0.0% | 0.0% |
| [8.0-9.0) | 1.1% | 1.1% | 1.1% |  | [8.0-9.0) | 5.56% | 5.56% | 5.56% |  | [8.0-9.0) | 0.0% | 0.0% | 0.0% |
| [9.0-10.0) | 0.7% | 0.7% | 0.7% |  | [9.0-10.0) | 4.35% | 4.35% | 4.35% |  | [9.0-10.0) | 0.0% | 0.0% | 0.0% |
| [10.0-11.0) | 1.6% | 1.0% | 0.5% |  | [10.0-11.0) | 8.82% | 5.88% | 2.94% |  | [10.0-11.0) | 0.0% | 0.0% | 0.0% |
| [11.0-12.0) | 3.0% | 3.0% | 1.9% |  | [11.0-12.0) | 10.87% | 8.70% | 6.52% |  | [11.0-12.0) | 1.4% | 1.8% | 0.9% |
| [12.0-13.0) | 4.9% | 4.3% | 2.3% |  | [12.0-13.0) | 10.94% | 7.81% | 6.25% |  | [12.0-13.0) | 3.5% | 3.5% | 1.4% |
| [13.0-14.0) | 6.3% | 5.9% | 2.9% |  | [13.0-14.0) | 13.25% | 9.64% | 7.23% |  | [13.0-14.0) | 4.7% | 5.0% | 1.9% |
| [14.0-15.0) | 7.0% | 6.4% | 3.5% |  | [14.0-15.0) | 14.56% | 10.68% | 8.74% |  | [14.0-15.0) | 5.2% | 5.5% | 2.3% |
| [15.0-16.0) | 7.3% | 7.0% | 4.4% |  | [15.0-16.0) | 15.57% | 12.30% | 10.66% |  | [15.0-16.0) | 5.4% | 5.8% | 3.0% |
| [16.0-17.0) | 8.2% | 8.1% | 4.9% |  | [16.0-17.0) | 16.88% | 14.94% | 11.04% |  | [16.0-17.0) | 6.0% | 6.3% | 3.4% |
| [17.0-18.0) | 9.6% | 9.1% | 5.5% |  | [17.0-18.0) | 19.46% | 16.76% | 11.89% |  | [17.0-18.0) | 6.9% | 7.1% | 3.8% |
| [18.0-19.0) | 10.9% | 10.7% | 6.9% |  | [18.0-19.0) | 22.27% | 20.45% | 14.55% |  | [18.0-19.0) | 7.7% | 7.9% | 4.7% |
| [19.0-20.0) | 12.4% | 12.2% | 8.0% |  | [19.0-20.0) | 24.28% | 22.63% | 16.05% |  | [19.0-20.0) | 9.0% | 9.2% | 5.6% |
| [20.0-21.0) | 13.7% | 13.7% | 8.9% |  | [20.0-21.0) | 26.62% | 25.54% | 17.27% |  | [20.0-21.0) | 9.8% | 10.1% | 6.4% |
| [21.0-22.0) | 15.7% | 15.3% | 9.3% |  | [21.0-22.0) | 27.83% | 26.30% | 16.21% |  | [21.0-22.0) | 11.7% | 11.6% | 7.0% |
| [22.0-23.0) | 16.3% | 16.1% | 9.6% |  | [22.0-23.0) | 28.53% | 27.40% | 16.38% |  | [22.0-23.0) | 12.3% | 12.4% | 7.3% |
| [23.0-24.0) | 17.5% | 17.1% | 9.9% |  | [23.0-24.0) | 29.87% | 28.35% | 17.22% |  | [23.0-24.0) | 13.3% | 13.4% | 7.4% |
| [24.0-25.0) | 18.6% | 18.5% | 10.3% |  | [24.0-25.0) | 31.28% | 29.68% | 16.89% |  | [24.0-25.0) | 14.1% | 14.5% | 7.9% |
| [25.0-26.0) | 19.6% | 19.4% | 10.3% |  | [25.0-26.0) | 31.73% | 30.48% | 16.49% |  | [25.0-26.0) | 15.0% | 15.3% | 8.1% |
| [26.0-27.0) | 20.6% | 20.0% | 10.7% |  | [26.0-27.0) | 33.02% | 30.57% | 16.79% |  | [26.0-27.0) | 15.7% | 16.0% | 8.4% |
| [27.0-28.0) | 21.8% | 21.1% | 11.2% |  | [27.0-28.0) | 34.16% | 31.15% | 16.64% |  | [27.0-28.0) | 16.9% | 17.2% | 9.0% |
| [28.0-29.0) | 22.7% | 22.0% | 11.7% |  | [28.0-29.0) | 35.40% | 32.05% | 17.79% |  | [28.0-29.0) | 17.7% | 18.1% | 9.4% |
| [29.0-30.0] | 23.5% | 22.5% | 11.8% |  | [29.0-30.0] | 36.60% | 32.77% | 17.46% |  | [29.0-30.0] | 18.2% | 18.4% | 9.6% |

| **Supplement Table 3: The detection rates of distant metastasis in T1-category lung cancer patients (M1a-M1c).** | | | | | | | | | | | | |  |
| --- | --- | --- | --- | --- | --- | --- | --- | --- | --- | --- | --- | --- | --- |
|  | **All** | | |  |  | **Solid** | | |  |  | **Sub-solid** | | |
| Diameter (mm) | M1a | M1b | M1c |  |  | M1a | M1b | M1c |  |  | M1a | M1b | M1c |
| [5.0-6.0) | 0.0% | 0.0% | 0.0% |  | [5.0-6.0) | 0.00% | 0.00% | 0.00% |  | [5.0-6.0) | 0.0% | 0.0% | 0.0% |
| [6.0-7.0) | 0.0% | 0.0% | 0.0% |  | [6.0-7.0) | 0.00% | 0.00% | 0.00% |  | [6.0-7.0) | 0.0% | 0.0% | 0.0% |
| [7.0-8.0) | 0.0% | 0.0% | 0.0% |  | [7.0-8.0) | 0.00% | 0.00% | 0.00% |  | [7.0-8.0) | 0.0% | 0.0% | 0.0% |
| [8.0-9.0) | 1.1% | 1.1% | 1.1% |  | [8.0-9.0) | 5.56% | 5.56% | 5.56% |  | [8.0-9.0) | 0.0% | 0.0% | 0.0% |
| [9.0-10.0) | 0.7% | 0.7% | 0.7% |  | [9.0-10.0) | 4.35% | 4.35% | 4.35% |  | [9.0-10.0) | 0.0% | 0.0% | 0.0% |
| [10.0-11.0) | 0.5% | 1.0% | 1.0% |  | [10.0-11.0) | 2.94% | 2.94% | 2.94% |  | [10.0-11.0) | 0.0% | 0.6% | 0.6% |
| [11.0-12.0) | 0.7% | 1.9% | 1.5% |  | [11.0-12.0) | 2.17% | 4.35% | 2.17% |  | [11.0-12.0) | 0.5% | 1.4% | 1.4% |
| [12.0-13.0) | 1.4% | 2.3% | 2.0% |  | [12.0-13.0) | 3.13% | 6.25% | 4.69% |  | [12.0-13.0) | 1.1% | 1.4% | 1.4% |
| [13.0-14.0) | 2.3% | 2.9% | 2.7% |  | [13.0-14.0) | 6.02% | 6.02% | 4.82% |  | [13.0-14.0) | 1.4% | 2.2% | 2.2% |
| [14.0-15.0) | 2.6% | 3.3% | 3.1% |  | [14.0-15.0) | 6.80% | 6.80% | 5.83% |  | [14.0-15.0) | 1.6% | 2.5% | 2.5% |
| [15.0-16.0) | 3.2% | 4.1% | 3.7% |  | [15.0-16.0) | 7.38% | 8.20% | 6.56% |  | [15.0-16.0) | 2.3% | 3.2% | 3.0% |
| [16.0-17.0) | 3.5% | 4.4% | 3.8% |  | [16.0-17.0) | 8.44% | 9.74% | 7.14% |  | [16.0-17.0) | 2.3% | 3.1% | 2.9% |
| [17.0-18.0) | 3.4% | 4.8% | 4.0% |  | [17.0-18.0) | 8.11% | 10.27% | 7.03% |  | [17.0-18.0) | 2.2% | 3.3% | 3.2% |
| [18.0-19.0) | 4.3% | 6.0% | 5.1% |  | [18.0-19.0) | 9.55% | 13.64% | 10.00% |  | [18.0-19.0) | 2.9% | 3.8% | 3.6% |
| [19.0-20.0) | 5.4% | 6.9% | 5.8% |  | [19.0-20.0) | 11.11% | 14.40% | 10.70% |  | [19.0-20.0) | 3.7% | 4.7% | 4.4% |
| [20.0-21.0) | 6.4% | 7.6% | 6.7% |  | [20.0-21.0) | 12.23% | 15.47% | 12.23% |  | [20.0-21.0) | 4.7% | 5.2% | 5.0% |
| [21.0-22.0) | 8.9% | 8.8% | 7.5% |  | [21.0-22.0) | 16.21% | 15.60% | 12.54% |  | [21.0-22.0) | 6.5% | 6.5% | 5.8% |
| [22.0-23.0) | 9.4% | 9.3% | 7.6% |  | [22.0-23.0) | 16.67% | 16.38% | 12.99% |  | [22.0-23.0) | 7.0% | 6.9% | 5.8% |
| [23.0-24.0) | 9.8% | 9.4% | 7.7% |  | [23.0-24.0) | 16.71% | 15.95% | 12.66% |  | [23.0-24.0) | 7.4% | 7.2% | 6.0% |
| [24.0-25.0) | 10.1% | 9.7% | 7.8% |  | [24.0-25.0) | 16.89% | 16.21% | 12.79% |  | [24.0-25.0) | 7.7% | 7.3% | 6.0% |
| [25.0-26.0) | 10.9% | 9.9% | 8.0% |  | [25.0-26.0) | 18.16% | 16.08% | 12.73% |  | [25.0-26.0) | 8.1% | 7.7% | 6.3% |
| [26.0-27.0) | 11.4% | 10.4% | 8.2% |  | [26.0-27.0) | 18.11% | 16.42% | 12.83% |  | [26.0-27.0) | 8.7% | 8.0% | 6.4% |
| [27.0-28.0) | 11.8% | 10.7% | 8.4% |  | [27.0-28.0) | 18.76% | 16.46% | 12.74% |  | [27.0-28.0) | 9.0% | 8.5% | 6.7% |
| [28.0-29.0) | 12.3% | 11.1% | 8.8% |  | [28.0-29.0) | 19.46% | 16.95% | 13.26% |  | [28.0-29.0) | 9.5% | 8.8% | 7.0% |
| [29.0-30.0] | 12.9% | 11.4% | 9.1% |  | [29.0-30.0] | 20.21% | 17.15% | 13.48% |  | [29.0-30.0] | 10.0% | 9.1% | 7.3% |

**Supplement Table 4: The demographic and clinical-pathological features of T1-category lung cancer patients with pulmonary nodules after propensity score matching.**

|  |  | **All** | |  | **Solid** | |  | **Sub-solid** | |  |
| --- | --- | --- | --- | --- | --- | --- | --- | --- | --- | --- |
|  |  | PET/CT  plus MRI | CT only  (or plus MRI) |  | PET/CT  plus MRI | CT only  (or plus MRI) |  | PET/CT  plus MRI | CT only  (or plus MRI) |  |
| Gender |  |  |  | P value |  |  | P value |  |  | P value |
|  | Male | 620 (50.7%) | 604 (49.3%) | 0.526 | 134 (57.0%) | 101 (43.0%) | 0.155 | 486 (49.1%) | 503 (50.9%) | 0.941 |
|  | Female | 654 (49.4%) | 670 (50.6%) |  | 90 (50.0%) | 90 (50.0%) |  | 564 (49.3%) | 580 (50.7%) |  |
| Age (year) |  |  |  |  |  |  |  |  |  |  |
|  | Median (IQR) | 59 (51-66) | 59 (51-66) | 0.454 | 62 (54-68) | 61 (54-68) | 0.748 | 59 (51-66) | 59 (51-65) | 0.626 |
| Nodule diameter (mm) | |  |  |  |  |  |  |  |  |  |
|  | Median (IQR) | 17.0 (13.0-22.0) | 17.0 (13.0-22.0) | 0.795 | 18.5 (14.0-23.0) | 20.0 (14.0-25.0) | 0.146 | 17.0 (13.0-22.0) | 17.0 (12.0-22.0) | 0.901 |
| Location |  |  |  |  |  |  |  |  |  |  |
|  | LUL | 358 (50.6%) | 349 (49.4%) | 0.988 | 69 (58.5%) | 49 (41.5%) | 0.058 | 289 (49.1%) | 300 (50.9%) | 0.745 |
|  | LLL | 149 (49.5%) | 152 (50.5%) |  | 41 (66.1%) | 21 (33.9%) |  | 108 (45.2%) | 131 (54.8%) |  |
|  | RUL | 478 (50.2%) | 474 (49.8%) |  | 66 (52.4%) | 60 (47.6%) |  | 412 (49.9%) | 414 (50.1%) |  |
|  | RML | 75 (49.0%) | 78 (51.0%) |  | 16 (43.2%) | 21 (56.8%) |  | 59 (50.9%) | 57 (49.1%) |  |
|  | RLL | 214 (49.2%) | 221 (50.8%) |  | 32 (44.4%) | 40 (55.6%) |  | 182 (50.1%) | 181 (49.9%) |  |
| Pathologic risk factors | |  |  |  |  |  |  |  |  |  |
| Pleural invasion |  |  |  |  |  |  |  |  |  |  |
|  | Yes | 143 (47.5%) | 158 (52.5%) | 0.357 | 53 (56.4%) | 41 (43.6%) | 0.594 | 90 (43.5%) | 117 (56.5%) | 0.082 |
|  | No | 1131 (50.3%) | 1116 (49.7%) |  | 171 (53.3%) | 150 (46.7%) |  | 960 (49.8%) | 966 (50.2%) |  |
| Vascular cancer embolus | |  |  |  |  |  |  |  |  |  |
|  | Yes | 94 (47.5%) | 104 (52.5%) | 0.459 | 43 (57.3%) | 32 (42.7%) | 0.519 | 51 (41.5%) | 72 (58.5%) | 0.076 |
|  | No | 1180 (50.2%) | 1170 (49.8%) |  | 181 (53.2%) | 159 (46.8%) |  | 999 (49.7%) | 1011 (50.3%) |  |
| Spread through air spaces | |  |  |  |  |  |  |  |  |  |
|  | Yes | 7 (53.8%) | 6 (46.2%) | 0.781 | 3 (60.0%) | 2 (40.0%) | 0.786 | 4 (50.0%) | 4 (50.0%) | 1.000 |
|  | No | 1267 (50.0%) | 1268 (50.0%) |  | 221 (53.9%) | 189 (46.1%) |  | 1046 (49.2%) | 1079 (50.8%) |  |
| Nerve invasion |  |  |  |  |  |  |  |  |  |  |
|  | Yes | 3 (33.3%) | 6 (66.7%) | 0.316 | 3 (75.0%) | 1 (25.0%) | 1.000 | 0 (0.0%) | 5 (100.0%) | 0.062 |
|  | No | 1271 (50.1%) | 1268 (49.9%) |  | 221 (53.8%) | 190 (46.2%) |  | 1050 (49.3%) | 1078 (50.7%) |  |
| Bronchial invasion |  |  |  |  |  |  |  |  |  |  |
|  | Yes | 17 (47.2%) | 19 (52.8%) | 0.737 | 6 (50.0%) | 6 (50.0%) | 0.779 | 11 (45.8%) | 13 (54.2%) | 0.738 |
|  | No | 1257 (50.0%) | 1255 (50.0%) |  | 218 (54.1%) | 185 (45.9%) |  | 1039 (49.3%) | 1070 (50.7%) |  |
| Vascular invasion |  |  |  |  |  |  |  |  |  |  |
|  | Yes | 3 (60.0%) | 2 (40.0%) | 0.654 | 2 (100.0%) | 0 (0.0%) | 0.502 | 1 (33.3%) | 2 (66.7%) | 1.000 |
|  | No | 1271 (50.0%) | 1272 (50.0%) |  | 222 (53.8%) | 191 (46.2%) |  | 1049 (49.2%) | 1081 (50.8%) |  |
| Histologic findings |  |  |  |  |  |  |  |  |  |  |
|  | LUAD | 1160 (50.0%) | 1159 (50.0%) | 0.948 | 186 (53.9%) | 159 (46.1%) | 0.818 | 974 (49.3%) | 1000 (50.7%) | 0.985 |
|  | LUSC | 44 (51.2%) | 42 (48.8%) |  | 22 (55.0%) | 18 (45.0%) |  | 22 (47.8%) | 24 (52.2%) |  |
|  | SCLC | 3 (60.0%) | 2 (40.0%) |  | 1 (100.0%) | 0 (0.0%) |  | 2 (50.0%) | 2 (50.0%) |  |
|  | Other | 67 (48.6%) | 71 (51.4%) |  | 15 (51.7%) | 14 (48.3%) |  | 52 (47.7%) | 57 (52.3%) |  |

Note： LUL, Left upper lobe; LLL, Left lower lobe; RUL, Right upper lobe; RML, Right middle lobe; RLL, Right lower lobe; LUAD, lung adenocarcinoma; LUSC, lung squamous cell carcinoma; SCLC, small cell lung cancer; AAH, Atypical adenomatous hyperplasia; AIS, Adenocarcinoma in situ; MIA, Minimally invasive adenocarcinoma; IA, Invasive adenocarcinoma; NA not available. *, High-grade patterns include solid, micropapillary, and complex glandular patterns.

**Supplement Table 5: The detection rates of metastasis in T1-category lung adenocarcinoma patients (overall metastasis, lymph node metastasis and distant metastasis).**

|  | **All** | | |  | **Solid** | | |  | **Sub-solid** | | |
| --- | --- | --- | --- | --- | --- | --- | --- | --- | --- | --- | --- |
| Diameter (mm) | Overall metastasis | Lymph node metastasis | Distant metastasis |  | Overall metastasis | Lymph node metastasis | Distant metastasis |  | Overall metastasis | Lymph node metastasis | Distant metastasis |
| [5.0-6.0) | 0.00% | 0.00% | 0.00% | [5.0-6.0) | 0.00% | 0.00% | 0.00% | [5.0-6.0) | 0.00% | 0.00% | 0.00% |
| [6.0-7.0) | 0.00% | 0.00% | 0.00% | [6.0-7.0) | 0.00% | 0.00% | 0.00% | [6.0-7.0) | 0.00% | 0.00% | 0.00% |
| [7.0-8.0) | 0.00% | 0.00% | 0.00% | [7.0-8.0) | 0.00% | 0.00% | 0.00% | [7.0-8.0) | 0.00% | 0.00% | 0.00% |
| [8.0-9.0) | 1.18% | 1.18% | 1.18% | [8.0-9.0) | 5.56% | 5.56% | 5.56% | [8.0-9.0) | 0.00% | 0.00% | 0.00% |
| [9.0-10.0) | 0.83% | 0.83% | 0.83% | [9.0-10.0) | 5.00% | 5.00% | 5.00% | [9.0-10.0) | 0.00% | 0.00% | 0.00% |
| [10.0-11.0) | 0.59% | 0.59% | 0.59% | [10.0-11.0) | 3.57% | 3.57% | 3.57% | [10.0-11.0) | 0.00% | 0.00% | 0.00% |
| [11.0-12.0) | 2.12% | 2.12% | 2.12% | [11.0-12.0) | 5.41% | 5.41% | 5.41% | [11.0-12.0) | 1.51% | 1.51% | 1.51% |
| [12.0-13.0) | 3.99% | 3.99% | 2.66% | [12.0-13.0) | 7.84% | 7.84% | 5.88% | [12.0-13.0) | 3.20% | 3.20% | 2.00% |
| [13.0-14.0) | 5.67% | 5.41% | 3.87% | [13.0-14.0) | 13.43% | 11.94% | 7.46% | [13.0-14.0) | 4.05% | 4.05% | 3.12% |
| [14.0-15.0) | 6.53% | 6.11% | 4.42% | [14.0-15.0) | 13.41% | 12.20% | 8.54% | [14.0-15.0) | 5.09% | 4.83% | 3.56% |
| [15.0-16.0) | 7.44% | 6.40% | 5.54% | [15.0-16.0) | 16.33% | 13.27% | 11.22% | [15.0-16.0) | 5.63% | 5.00% | 4.38% |
| [16.0-17.0) | 8.64% | 7.45% | 5.96% | [16.0-17.0) | 19.67% | 16.39% | 13.93% | [16.0-17.0) | 6.19% | 5.46% | 4.19% |
| [17.0-18.0) | 9.51% | 8.32% | 6.21% | [17.0-18.0) | 21.13% | 17.61% | 14.79% | [17.0-18.0) | 6.83% | 6.18% | 4.23% |
| [18.0-19.0) | 11.08% | 9.79% | 7.31% | [18.0-19.0) | 24.86% | 21.39% | 16.76% | [18.0-19.0) | 7.56% | 6.81% | 4.89% |
| [19.0-20.0) | 12.69% | 11.17% | 8.57% | [19.0-20.0) | 27.13% | 23.40% | 18.62% | [19.0-20.0) | 8.99% | 8.04% | 5.99% |
| [20.0-21.0) | 14.61% | 12.76% | 10.03% | [20.0-21.0) | 30.28% | 26.15% | 21.10% | [20.0-21.0) | 10.38% | 9.15% | 7.05% |
| [21.0-22.0) | 17.45% | 14.79% | 12.75% | [21.0-22.0) | 33.46% | 26.92% | 25.00% | [21.0-22.0) | 12.66% | 11.16% | 9.09% |
| [22.0-23.0) | 18.18% | 15.12% | 13.47% | [22.0-23.0) | 34.75% | 26.95% | 26.24% | [22.0-23.0) | 13.15% | 11.53% | 9.59% |
| [23.0-24.0) | 19.47% | 16.36% | 14.09% | [23.0-24.0) | 35.35% | 28.03% | 26.11% | [23.0-24.0) | 14.51% | 12.72% | 10.34% |
| [24.0-25.0) | 20.93% | 17.57% | 14.57% | [24.0-25.0) | 37.43% | 29.24% | 26.61% | [24.0-25.0) | 15.60% | 13.80% | 10.68% |
| [25.0-26.0) | 22.21% | 18.64% | 15.46% | [25.0-26.0) | 38.67% | 30.13% | 27.73% | [25.0-26.0) | 16.64% | 14.74% | 11.30% |
| [26.0-27.0) | 23.18% | 19.27% | 16.05% | [26.0-27.0) | 39.09% | 30.46% | 27.82% | [26.0-27.0) | 17.50% | 15.27% | 11.84% |
| [27.0-28.0) | 24.55% | 20.33% | 16.71% | [27.0-28.0) | 40.41% | 31.28% | 28.77% | [27.0-28.0) | 18.85% | 16.39% | 12.38% |
| [28.0-29.0) | 25.76% | 21.19% | 17.42% | [28.0-29.0) | 41.52% | 32.17% | 29.35% | [28.0-29.0) | 20.14% | 17.27% | 13.17% |
| [29.0-30.0] | 26.64% | 22.04% | 18.08% | [29.0-30.0] | 43.25% | 33.93% | 30.16% | [29.0-30.0] | 20.61% | 17.72% | 13.69% |

| **Supplement Table 6: The detection rates of lymph node metastasis in T1-category lung adenocarcinoma patients (N1-N3).** | | | | | | | | | | | | | |
| --- | --- | --- | --- | --- | --- | --- | --- | --- | --- | --- | --- | --- | --- |
|  | **All** | | |  |  | **Solid** | | |  |  | **Sub-solid** | | |
| Diameter (mm) | N1 | N2 | N3 |  |  | N1 | N2 | N3 |  |  | N1 | N2 | N3 |
| [5.0-6.0) | 0.00% | 0.00% | 0.00% |  | [5.0-6.0) | 0.00% | 0.00% | 0.00% |  | [5.0-6.0) | 0.00% | 0.00% | 0.00% |
| [6.0-7.0) | 0.00% | 0.00% | 0.00% |  | [6.0-7.0) | 0.00% | 0.00% | 0.00% |  | [6.0-7.0) | 0.00% | 0.00% | 0.00% |
| [7.0-8.0) | 0.00% | 0.00% | 0.00% |  | [7.0-8.0) | 0.00% | 0.00% | 0.00% |  | [7.0-8.0) | 0.00% | 0.00% | 0.00% |
| [8.0-9.0) | 1.18% | 1.18% | 1.18% |  | [8.0-9.0) | 5.56% | 5.56% | 5.56% |  | [8.0-9.0) | 0.00% | 0.00% | 0.00% |
| [9.0-10.0) | 0.83% | 0.83% | 0.83% |  | [9.0-10.0) | 5.00% | 5.00% | 5.00% |  | [9.0-10.0) | 0.00% | 0.00% | 0.00% |
| [10.0-11.0) | 0.59% | 0.59% | 0.59% |  | [10.0-11.0) | 3.57% | 3.57% | 3.57% |  | [10.0-11.0) | 0.00% | 0.00% | 0.00% |
| [11.0-12.0) | 1.69% | 2.12% | 1.27% |  | [11.0-12.0) | 5.41% | 5.41% | 5.41% |  | [11.0-12.0) | 1.01% | 1.51% | 0.50% |
| [12.0-13.0) | 3.65% | 3.32% | 1.99% |  | [12.0-13.0) | 7.84% | 5.88% | 5.88% |  | [12.0-13.0) | 2.80% | 2.80% | 1.20% |
| [13.0-14.0) | 4.90% | 4.64% | 2.58% |  | [13.0-14.0) | 10.45% | 8.96% | 5.97% |  | [13.0-14.0) | 3.74% | 3.74% | 1.87% |
| [14.0-15.0) | 5.47% | 5.05% | 2.95% |  | [14.0-15.0) | 10.98% | 8.54% | 6.10% |  | [14.0-15.0) | 4.33% | 4.33% | 2.29% |
| [15.0-16.0) | 5.88% | 5.36% | 3.46% |  | [15.0-16.0) | 12.24% | 9.18% | 7.14% |  | [15.0-16.0) | 4.58% | 4.58% | 2.71% |
| [16.0-17.0) | 6.41% | 6.26% | 4.02% |  | [16.0-17.0) | 13.93% | 12.30% | 9.02% |  | [16.0-17.0) | 4.74% | 4.92% | 2.91% |
| [17.0-18.0) | 7.40% | 7.13% | 4.49% |  | [17.0-18.0) | 15.49% | 13.38% | 9.15% |  | [17.0-18.0) | 5.53% | 5.69% | 3.41% |
| [18.0-19.0) | 8.84% | 8.73% | 5.78% |  | [18.0-19.0) | 19.08% | 17.92% | 12.14% |  | [18.0-19.0) | 6.22% | 6.37% | 4.15% |
| [19.0-20.0) | 10.20% | 10.20% | 7.05% |  | [19.0-20.0) | 20.74% | 20.21% | 13.83% |  | [19.0-20.0) | 7.49% | 7.63% | 5.31% |
| [20.0-21.0) | 11.68% | 11.78% | 8.08% |  | [20.0-21.0) | 23.85% | 23.39% | 15.60% |  | [20.0-21.0) | 8.41% | 8.65% | 6.06% |
| [21.0-22.0) | 13.91% | 13.46% | 8.33% |  | [21.0-22.0) | 25.77% | 24.23% | 14.62% |  | [21.0-22.0) | 10.36% | 10.24% | 6.44% |
| [22.0-23.0) | 14.38% | 13.97% | 8.51% |  | [22.0-23.0) | 26.24% | 24.82% | 14.54% |  | [22.0-23.0) | 10.78% | 10.67% | 6.68% |
| [23.0-24.0) | 15.53% | 15.00% | 9.09% |  | [23.0-24.0) | 27.39% | 25.80% | 15.61% |  | [23.0-24.0) | 11.83% | 11.63% | 7.06% |
| [24.0-25.0) | 16.71% | 16.29% | 9.50% |  | [24.0-25.0) | 29.24% | 27.19% | 15.50% |  | [24.0-25.0) | 12.67% | 12.76% | 7.56% |
| [25.0-26.0) | 17.69% | 17.35% | 9.72% |  | [25.0-26.0) | 29.60% | 28.27% | 15.47% |  | [25.0-26.0) | 13.65% | 13.65% | 7.78% |
| [26.0-27.0) | 18.45% | 17.62% | 9.92% |  | [26.0-27.0) | 30.46% | 28.06% | 15.59% |  | [26.0-27.0) | 14.15% | 13.89% | 7.89% |
| [27.0-28.0) | 19.42% | 18.70% | 10.37% |  | [27.0-28.0) | 31.05% | 28.77% | 15.98% |  | [27.0-28.0) | 15.25% | 15.08% | 8.36% |
| [28.0-29.0) | 20.22% | 19.47% | 10.91% |  | [28.0-29.0) | 32.17% | 29.78% | 17.17% |  | [28.0-29.0) | 15.96% | 15.80% | 8.68% |
| [29.0-30.0] | 20.88% | 20.03% | 11.15% |  | [29.0-30.0] | 33.53% | 30.95% | 17.06% |  | [29.0-30.0] | 16.28% | 16.07% | 9.01% |

**Supplement Table 7: The detection rates of distant metastasis in T1-category lung adenocarcinoma patients (M1a-M1c).**

|  | **All** | | |  | **Solid** | | |  | **Sub-solid** | | |
| --- | --- | --- | --- | --- | --- | --- | --- | --- | --- | --- | --- |
| Diameter (mm) | M1a | M1b | M1c |  | M1a | M1b | M1c |  | M1a | M1b | M1c |
| [5.0-6.0) | 0.00% | 0.00% | 0.00% | [5.0-6.0) | 0.00% | 0.00% | 0.00% | [5.0-6.0) | 0.00% | 0.00% | 0.00% |
| [6.0-7.0) | 0.00% | 0.00% | 0.00% | [6.0-7.0) | 0.00% | 0.00% | 0.00% | [6.0-7.0) | 0.00% | 0.00% | 0.00% |
| [7.0-8.0) | 0.00% | 0.00% | 0.00% | [7.0-8.0) | 0.00% | 0.00% | 0.00% | [7.0-8.0) | 0.00% | 0.00% | 0.00% |
| [8.0-9.0) | 1.18% | 1.18% | 1.18% | [8.0-9.0) | 5.56% | 5.56% | 5.56% | [8.0-9.0) | 0.00% | 0.00% | 0.00% |
| [9.0-10.0) | 0.83% | 0.83% | 0.83% | [9.0-10.0) | 5.00% | 5.00% | 5.00% | [9.0-10.0) | 0.00% | 0.00% | 0.00% |
| [10.0-11.0) | 1.18% | 0.59% | 0.59% | [10.0-11.0) | 3.57% | 3.57% | 3.57% | [10.0-11.0) | 0.71% | 0.00% | 0.00% |
| [11.0-12.0) | 1.69% | 1.69% | 1.27% | [11.0-12.0) | 2.70% | 5.41% | 2.70% | [11.0-12.0) | 1.51% | 1.01% | 1.01% |
| [12.0-13.0) | 1.99% | 1.99% | 1.66% | [12.0-13.0) | 1.96% | 5.88% | 3.92% | [12.0-13.0) | 2.00% | 1.20% | 1.20% |
| [13.0-14.0) | 2.58% | 2.58% | 2.32% | [13.0-14.0) | 4.48% | 4.48% | 2.99% | [13.0-14.0) | 2.18% | 2.18% | 2.18% |
| [14.0-15.0) | 3.37% | 2.95% | 2.74% | [14.0-15.0) | 6.10% | 4.88% | 3.66% | [14.0-15.0) | 2.80% | 2.54% | 2.54% |
| [15.0-16.0) | 3.29% | 3.81% | 3.29% | [15.0-16.0) | 7.14% | 7.14% | 5.10% | [15.0-16.0) | 2.50% | 3.13% | 2.92% |
| [16.0-17.0) | 3.43% | 4.17% | 3.43% | [16.0-17.0) | 8.20% | 9.84% | 6.56% | [16.0-17.0) | 2.37% | 2.91% | 2.73% |
| [17.0-18.0) | 4.10% | 4.49% | 3.57% | [17.0-18.0) | 7.75% | 11.27% | 7.04% | [17.0-18.0) | 3.25% | 2.93% | 2.76% |
| [18.0-19.0) | 5.31% | 5.54% | 4.60% | [18.0-19.0) | 9.83% | 13.87% | 9.83% | [18.0-19.0) | 4.15% | 3.41% | 3.26% |
| [19.0-20.0) | 6.62% | 6.62% | 5.53% | [19.0-20.0) | 11.70% | 14.89% | 10.64% | [19.0-20.0) | 5.31% | 4.50% | 4.22% |
| [20.0-21.0) | 7.69% | 7.40% | 6.43% | [20.0-21.0) | 13.30% | 16.51% | 12.84% | [20.0-21.0) | 4.82% | 4.94% | 4.70% |
| [21.0-22.0) | 10.27% | 8.77% | 7.35% | [21.0-22.0) | 17.69% | 16.54% | 13.08% | [21.0-22.0) | 6.79% | 6.44% | 5.64% |
| [22.0-23.0) | 10.74% | 9.34% | 7.52% | [22.0-23.0) | 18.09% | 17.38% | 13.48% | [22.0-23.0) | 7.33% | 6.90% | 5.71% |
| [23.0-24.0) | 11.29% | 9.47% | 7.65% | [23.0-24.0) | 18.47% | 17.20% | 13.38% | [23.0-24.0) | 7.95% | 7.06% | 5.86% |
| [24.0-25.0) | 11.64% | 9.71% | 7.79% | [24.0-25.0) | 18.71% | 17.25% | 13.45% | [24.0-25.0) | 8.32% | 7.28% | 5.95% |
| [25.0-26.0) | 12.42% | 10.20% | 8.17% | [25.0-26.0) | 20.27% | 17.33% | 13.60% | [25.0-26.0) | 8.77% | 7.78% | 6.33% |
| [26.0-27.0) | 12.89% | 10.49% | 8.34% | [26.0-27.0) | 20.38% | 17.75% | 13.91% | [26.0-27.0) | 9.26% | 7.89% | 6.35% |
| [27.0-28.0) | 13.27% | 10.80% | 8.44% | [27.0-28.0) | 21.46% | 18.04% | 14.16% | [27.0-28.0) | 9.43% | 8.20% | 6.39% |
| [28.0-29.0) | 13.88% | 11.19% | 8.91% | [28.0-29.0) | 22.39% | 18.48% | 14.57% | [28.0-29.0) | 9.99% | 8.60% | 6.89% |
| [29.0-30.0] | 14.59% | 11.42% | 9.14% | [29.0-30.0] | 23.41% | 18.65% | 14.68% | [29.0-30.0] | 10.59% | 8.79% | 7.13% |

| **Supplement Table 8: The detection rates of metastasis in T1-category non-adenocarcinoma lung cancer patients (overall metastasis, lymph node metastasis and distant metastasis).** | | | | | | | | | | | |  |  |  |
| --- | --- | --- | --- | --- | --- | --- | --- | --- | --- | --- | --- | --- | --- | --- |
|  | **All** | | |  | **Solid** | | |  | **Sub-solid** | | |  |  |  |
| Diameter (mm) | Overall metastasis | Lymph node metastasis | Distant metastasis |  | Overall metastasis | Lymph node metastasis | Distant metastasis |  | Overall metastasis | Lymph node metastasis | Distant metastasis |  |  |  |
| [5.0-6.0) | 0.00% | 0.00% | 0.00% | [5.0-6.0) | 0.00% | 0.00% | 0.00% | [5.0-6.0) | 0.00% | 0.00% | 0.00% |  |  |  |
| [6.0-7.0) | 0.00% | 0.00% | 0.00% | [6.0-7.0) | 0.00% | 0.00% | 0.00% | [6.0-7.0) | 0.00% | 0.00% | 0.00% |  |  |  |
| [7.0-8.0) | 0.00% | 0.00% | 0.00% | [7.0-8.0) | 0.00% | 0.00% | 0.00% | [7.0-8.0) | 0.00% | 0.00% | 0.00% |  |  |  |
| [8.0-9.0) | 0.00% | 0.00% | 0.00% | [8.0-9.0) | 0.00% | 0.00% | 0.00% | [8.0-9.0) | 0.00% | 0.00% | 0.00% |  |  |  |
| [9.0-10.0) | 0.00% | 0.00% | 0.00% | [9.0-10.0) | 0.00% | 0.00% | 0.00% | [9.0-10.0) | 0.00% | 0.00% | 0.00% |  |  |  |
| [10.0-11.0) | 12.50% | 8.33% | 0.00% | [10.0-11.0) | 33.33% | 33.33% | 0.00% | [10.0-11.0) | 5.56% | 0.00% | 0.00% |  |  |  |
| [11.0-12.0) | 16.13% | 12.90% | 3.23% | [11.0-12.0) | 33.33% | 33.33% | 0.00% | [11.0-12.0) | 9.09% | 4.55% | 4.55% |  |  |  |
| [12.0-13.0) | 17.39% | 13.04% | 4.35% | [12.0-13.0) | 30.77% | 23.08% | 7.69% | [12.0-13.0) | 12.12% | 9.09% | 3.03% |  |  |  |
| [13.0-14.0) | 21.82% | 16.36% | 5.45% | [13.0-14.0) | 31.25% | 25.00% | 12.50% | [13.0-14.0) | 17.95% | 12.82% | 2.56% |  |  |  |
| [14.0-15.0) | 22.06% | 17.65% | 5.88% | [14.0-15.0) | 33.33% | 28.57% | 14.29% | [14.0-15.0) | 17.02% | 12.77% | 2.13% |  |  |  |
| [15.0-16.0) | 24.68% | 20.78% | 6.49% | [15.0-16.0) | 37.50% | 33.33% | 12.50% | [15.0-16.0) | 18.87% | 15.09% | 3.77% |  |  |  |
| [16.0-17.0) | 25.25% | 22.22% | 7.07% | [16.0-17.0) | 34.38% | 31.25% | 12.50% | [16.0-17.0) | 20.90% | 17.91% | 4.48% |  |  |  |
| [17.0-18.0) | 27.73% | 25.21% | 8.40% | [17.0-18.0) | 37.21% | 34.88% | 11.63% | [17.0-18.0) | 22.37% | 19.74% | 6.58% |  |  |  |
| [18.0-19.0) | 29.08% | 26.24% | 9.93% | [18.0-19.0) | 40.43% | 36.17% | 17.02% | [18.0-19.0) | 23.40% | 21.28% | 6.38% |  |  |  |
| [19.0-20.0) | 30.57% | 28.03% | 10.83% | [19.0-20.0) | 41.82% | 38.18% | 18.18% | [19.0-20.0) | 24.51% | 22.55% | 6.86% |  |  |  |
| [20.0-21.0) | 31.03% | 28.16% | 10.92% | [20.0-21.0) | 43.33% | 40.00% | 16.67% | [20.0-21.0) | 24.56% | 21.93% | 7.89% |  |  |  |
| [21.0-22.0) | 31.79% | 29.23% | 11.79% | [21.0-22.0) | 43.28% | 40.30% | 17.91% | [21.0-22.0) | 25.78% | 23.44% | 8.59% |  |  |  |
| [22.0-23.0) | 35.55% | 32.23% | 12.32% | [22.0-23.0) | 47.22% | 43.06% | 18.06% | [22.0-23.0) | 29.50% | 26.62% | 9.35% |  |  |  |
| [23.0-24.0) | 36.21% | 32.92% | 11.93% | [23.0-24.0) | 48.15% | 44.44% | 16.05% | [23.0-24.0) | 30.25% | 27.16% | 9.88% |  |  |  |
| [24.0-25.0) | 37.31% | 33.58% | 12.31% | [24.0-25.0) | 48.96% | 43.75% | 17.71% | [24.0-25.0) | 30.81% | 27.91% | 9.30% |  |  |  |
| [25.0-26.0) | 38.19% | 34.38% | 12.15% | [25.0-26.0) | 50.96% | 45.19% | 17.31% | [25.0-26.0) | 30.98% | 28.26% | 9.24% |  |  |  |
| [26.0-27.0) | 41.07% | 36.99% | 13.48% | [26.0-27.0) | 53.10% | 47.79% | 16.81% | [26.0-27.0) | 34.47% | 31.07% | 11.65% |  |  |  |
| [27.0-28.0) | 42.57% | 38.00% | 14.00% | [27.0-28.0) | 54.33% | 48.82% | 16.54% | [27.0-28.0) | 35.87% | 31.84% | 12.56% |  |  |  |
| [28.0-29.0) | 43.85% | 39.57% | 14.17% | [28.0-29.0) | 55.15% | 50.00% | 16.91% | [28.0-29.0) | 37.39% | 33.61% | 12.61% |  |  |  |
| [29.0-30.0] | 44.83% | 40.39% | 14.53% | [29.0-30.0] | 55.70% | 49.66% | 16.78% | [29.0-30.0] | 38.52% | 35.02% | 13.23% |  |  |  |

| **Supplement Table 9: The detection rates of lymph node metastasis in T1-category non-adenocarcinoma lung cancer patients (N1-N3).** | | | | | | | | | | | | | |
| --- | --- | --- | --- | --- | --- | --- | --- | --- | --- | --- | --- | --- | --- |
|  | **All** | | |  |  | **Solid** | | |  |  | **Sub-solid** | | |
| Diameter (mm) | N1 | N2 | N3 |  |  | N1 | N2 | N3 |  |  | N1 | N2 | N3 |
| [5.0-6.0) | 0.00% | 0.00% | 0.00% |  | [5.0-6.0) | 0.00% | 0.00% | 0.00% |  | [5.0-6.0) | 0.00% | 0.00% | 0.00% |
| [6.0-7.0) | 0.00% | 0.00% | 0.00% |  | [6.0-7.0) | 0.00% | 0.00% | 0.00% |  | [6.0-7.0) | 0.00% | 0.00% | 0.00% |
| [7.0-8.0) | 0.00% | 0.00% | 0.00% |  | [7.0-8.0) | 0.00% | 0.00% | 0.00% |  | [7.0-8.0) | 0.00% | 0.00% | 0.00% |
| [8.0-9.0) | 0.00% | 0.00% | 0.00% |  | [8.0-9.0) | 0.00% | 0.00% | 0.00% |  | [8.0-9.0) | 0.00% | 0.00% | 0.00% |
| [9.0-10.0) | 0.00% | 0.00% | 0.00% |  | [9.0-10.0) | 0.00% | 0.00% | 0.00% |  | [9.0-10.0) | 0.00% | 0.00% | 0.00% |
| [10.0-11.0) | 8.33% | 4.17% | 0.00% |  | [10.0-11.0) | 33.33% | 16.67% | 0.00% |  | [10.0-11.0) | 0.00% | 0.00% | 0.00% |
| [11.0-12.0) | 12.90% | 9.68% | 6.45% |  | [11.0-12.0) | 33.33% | 22.22% | 11.11% |  | [11.0-12.0) | 4.55% | 4.55% | 4.55% |
| [12.0-13.0) | 13.04% | 10.87% | 4.35% |  | [12.0-13.0) | 23.08% | 15.38% | 7.69% |  | [12.0-13.0) | 9.09% | 9.09% | 3.03% |
| [13.0-14.0) | 16.36% | 14.55% | 5.45% |  | [13.0-14.0) | 25.00% | 12.50% | 12.50% |  | [13.0-14.0) | 12.82% | 15.38% | 2.56% |
| [14.0-15.0) | 17.65% | 16.18% | 7.35% |  | [14.0-15.0) | 28.57% | 19.05% | 19.05% |  | [14.0-15.0) | 12.77% | 14.89% | 2.13% |
| [15.0-16.0) | 18.18% | 19.48% | 11.69% |  | [15.0-16.0) | 29.17% | 25.00% | 25.00% |  | [15.0-16.0) | 13.21% | 16.98% | 5.66% |
| [16.0-17.0) | 20.20% | 20.20% | 11.11% |  | [16.0-17.0) | 28.13% | 25.00% | 18.75% |  | [16.0-17.0) | 16.42% | 17.91% | 7.46% |
| [17.0-18.0) | 23.53% | 21.85% | 11.76% |  | [17.0-18.0) | 32.56% | 27.91% | 20.93% |  | [17.0-18.0) | 18.42% | 18.42% | 6.58% |
| [18.0-19.0) | 23.40% | 22.70% | 13.48% |  | [18.0-19.0) | 34.04% | 29.79% | 23.40% |  | [18.0-19.0) | 18.09% | 19.15% | 8.51% |
| [19.0-20.0) | 25.48% | 24.20% | 13.38% |  | [19.0-20.0) | 36.36% | 30.91% | 23.64% |  | [19.0-20.0) | 19.61% | 20.59% | 7.84% |
| [20.0-21.0) | 25.29% | 24.71% | 13.79% |  | [20.0-21.0) | 36.67% | 33.33% | 23.33% |  | [20.0-21.0) | 19.30% | 20.18% | 8.77% |
| [21.0-22.0) | 26.15% | 25.64% | 14.87% |  | [21.0-22.0) | 35.82% | 34.33% | 22.39% |  | [21.0-22.0) | 21.09% | 21.09% | 10.94% |
| [22.0-23.0) | 27.49% | 28.44% | 15.64% |  | [22.0-23.0) | 37.50% | 37.50% | 23.61% |  | [22.0-23.0) | 22.30% | 23.74% | 11.51% |
| [23.0-24.0) | 27.98% | 28.81% | 14.40% |  | [23.0-24.0) | 39.51% | 38.27% | 23.46% |  | [23.0-24.0) | 22.22% | 24.07% | 9.88% |
| [24.0-25.0) | 28.73% | 29.85% | 14.18% |  | [24.0-25.0) | 38.54% | 38.54% | 21.88% |  | [24.0-25.0) | 23.26% | 25.00% | 9.88% |
| [25.0-26.0) | 29.17% | 29.86% | 13.54% |  | [25.0-26.0) | 39.42% | 38.46% | 20.19% |  | [25.0-26.0) | 23.37% | 25.00% | 9.78% |
| [26.0-27.0) | 31.03% | 31.97% | 14.73% |  | [26.0-27.0) | 42.48% | 39.82% | 21.24% |  | [26.0-27.0) | 24.76% | 27.67% | 11.17% |
| [27.0-28.0) | 32.86% | 32.57% | 14.86% |  | [27.0-28.0) | 44.88% | 39.37% | 18.90% |  | [27.0-28.0) | 26.01% | 28.70% | 12.56% |
| [28.0-29.0) | 34.22% | 33.96% | 15.51% |  | [28.0-29.0) | 46.32% | 39.71% | 19.85% |  | [28.0-29.0) | 27.31% | 30.67% | 13.03% |
| [29.0-30.0] | 35.47% | 33.74% | 15.02% |  | [29.0-30.0] | 46.98% | 38.93% | 18.79% |  | [29.0-30.0] | 28.79% | 30.74% | 12.84% |

| **Supplement Table 10: The detection rates of distant metastasis in T1-category non-adenocarcinoma lung cancer patients (M1a-M1c).** | | | | | | | | | | | | | |
| --- | --- | --- | --- | --- | --- | --- | --- | --- | --- | --- | --- | --- | --- |
|  | **All** | | |  |  | **Solid** | | |  |  | **Sub-solid** | | |
| Diameter (mm) | M1a | M1b | M1c |  |  | M1a | M1b | M1c |  |  | M1a | M1b | M1c |
| [5.0-6.0) | 0.00% | 0.00% | 0.00% |  | [5.0-6.0) | 0.00% | 0.00% | 0.00% |  | [5.0-6.0) | 0.00% | 0.00% | 0.00% |
| [6.0-7.0) | 0.00% | 0.00% | 0.00% |  | [6.0-7.0) | 0.00% | 0.00% | 0.00% |  | [6.0-7.0) | 0.00% | 0.00% | 0.00% |
| [7.0-8.0) | 0.00% | 0.00% | 0.00% |  | [7.0-8.0) | 0.00% | 0.00% | 0.00% |  | [7.0-8.0) | 0.00% | 0.00% | 0.00% |
| [8.0-9.0) | 0.00% | 0.00% | 0.00% |  | [8.0-9.0) | 0.00% | 0.00% | 0.00% |  | [8.0-9.0) | 0.00% | 0.00% | 0.00% |
| [9.0-10.0) | 0.00% | 0.00% | 0.00% |  | [9.0-10.0) | 0.00% | 0.00% | 0.00% |  | [9.0-10.0) | 0.00% | 0.00% | 0.00% |
| [10.0-11.0) | 0.00% | 4.17% | 4.17% |  | [10.0-11.0) | 0.00% | 0.00% | 0.00% |  | [10.0-11.0) | 0.00% | 5.56% | 5.56% |
| [11.0-12.0) | 0.00% | 3.23% | 3.23% |  | [11.0-12.0) | 0.00% | 0.00% | 0.00% |  | [11.0-12.0) | 0.00% | 4.55% | 4.55% |
| [12.0-13.0) | 2.17% | 4.35% | 4.35% |  | [12.0-13.0) | 7.69% | 7.69% | 7.69% |  | [12.0-13.0) | 0.00% | 3.03% | 3.03% |
| [13.0-14.0) | 3.64% | 5.45% | 5.45% |  | [13.0-14.0) | 12.50% | 12.50% | 12.50% |  | [13.0-14.0) | 0.00% | 2.56% | 2.56% |
| [14.0-15.0) | 4.41% | 5.88% | 5.88% |  | [14.0-15.0) | 9.52% | 14.29% | 14.29% |  | [14.0-15.0) | 2.13% | 2.13% | 2.13% |
| [15.0-16.0) | 5.19% | 6.49% | 6.49% |  | [15.0-16.0) | 8.33% | 12.50% | 12.50% |  | [15.0-16.0) | 3.77% | 3.77% | 3.77% |
| [16.0-17.0) | 5.05% | 6.06% | 6.06% |  | [16.0-17.0) | 9.38% | 9.38% | 9.38% |  | [16.0-17.0) | 2.99% | 4.48% | 4.48% |
| [17.0-18.0) | 5.04% | 6.72% | 6.72% |  | [17.0-18.0) | 9.30% | 6.98% | 6.98% |  | [17.0-18.0) | 2.63% | 6.58% | 6.58% |
| [18.0-19.0) | 4.96% | 8.51% | 7.80% |  | [18.0-19.0) | 8.51% | 12.77% | 10.64% |  | [18.0-19.0) | 3.19% | 6.38% | 6.38% |
| [19.0-20.0) | 5.73% | 8.28% | 7.64% |  | [19.0-20.0) | 9.09% | 12.73% | 10.91% |  | [19.0-20.0) | 3.92% | 5.88% | 5.88% |
| [20.0-21.0) | 5.75% | 8.62% | 8.05% |  | [20.0-21.0) | 8.33% | 11.67% | 10.00% |  | [20.0-21.0) | 3.51% | 7.02% | 7.02% |
| [21.0-22.0) | 7.18% | 8.72% | 8.21% |  | [21.0-22.0) | 10.45% | 11.94% | 10.45% |  | [21.0-22.0) | 4.69% | 7.03% | 7.03% |
| [22.0-23.0) | 7.58% | 9.00% | 8.06% |  | [22.0-23.0) | 11.11% | 12.50% | 11.11% |  | [22.0-23.0) | 5.04% | 7.19% | 6.47% |
| [23.0-24.0) | 6.58% | 9.05% | 7.82% |  | [23.0-24.0) | 9.88% | 11.11% | 9.88% |  | [23.0-24.0) | 4.32% | 8.02% | 6.79% |
| [24.0-25.0) | 6.72% | 9.33% | 7.84% |  | [24.0-25.0) | 10.42% | 12.50% | 10.42% |  | [24.0-25.0) | 4.07% | 7.56% | 6.40% |
| [25.0-26.0) | 6.94% | 8.68% | 7.29% |  | [25.0-26.0) | 10.58% | 11.54% | 9.62% |  | [25.0-26.0) | 4.35% | 7.07% | 5.98% |
| [26.0-27.0) | 7.52% | 9.72% | 7.52% |  | [26.0-27.0) | 9.73% | 11.50% | 8.85% |  | [26.0-27.0) | 5.83% | 8.74% | 6.80% |
| [27.0-28.0) | 8.00% | 10.29% | 8.00% |  | [27.0-28.0) | 9.45% | 11.02% | 7.87% |  | [27.0-28.0) | 6.73% | 9.87% | 8.07% |
| [28.0-29.0) | 8.29% | 10.43% | 8.02% |  | [28.0-29.0) | 9.56% | 11.76% | 8.82% |  | [28.0-29.0) | 7.14% | 9.66% | 7.56% |
| [29.0-30.0] | 8.13% | 11.08% | 8.62% |  | [29.0-30.0] | 9.40% | 12.08% | 9.40% |  | [29.0-30.0] | 7.00% | 10.51% | 8.17% |

| **Supplement Table 11: The detection rates of metastasis in T1-category lung cancer patients with EGFR mutation (overall metastasis, lymph node metastasis and distant metastasis).** | | | | | | | | | | | |  |  |  |
| --- | --- | --- | --- | --- | --- | --- | --- | --- | --- | --- | --- | --- | --- | --- |
|  | **All** | | |  | **Solid** | | |  | **Sub-solid** | | |  |  |  |
| Diameter (mm) | Overall metastasis | Lymph node metastasis | Distant metastasis |  | Overall metastasis | Lymph node metastasis | Distant metastasis |  | Overall metastasis | Lymph node metastasis | Distant metastasis |  |  |  |
| [5.0-6.0) | 0.00% | 0.00% | 0.00% | [5.0-6.0) | 0.00% | 0.00% | 0.00% | [5.0-6.0) | 0.00% | 0.00% | 0.00% |  |  |  |
| [6.0-7.0) | 0.00% | 0.00% | 0.00% | [6.0-7.0) | 0.00% | 0.00% | 0.00% | [6.0-7.0) | 0.00% | 0.00% | 0.00% |  |  |  |
| [7.0-8.0) | 0.00% | 0.00% | 0.00% | [7.0-8.0) | 0.00% | 0.00% | 0.00% | [7.0-8.0) | 0.00% | 0.00% | 0.00% |  |  |  |
| [8.0-9.0) | 0.00% | 0.00% | 0.00% | [8.0-9.0) | 0.00% | 0.00% | 0.00% | [8.0-9.0) | 0.00% | 0.00% | 0.00% |  |  |  |
| [9.0-10.0) | 0.00% | 0.00% | 0.00% | [9.0-10.0) | 0.00% | 0.00% | 0.00% | [9.0-10.0) | 0.00% | 0.00% | 0.00% |  |  |  |
| [10.0-11.0) | 0.00% | 0.00% | 0.00% | [10.0-11.0) | 0.00% | 0.00% | 0.00% | [10.0-11.0) | 0.00% | 0.00% | 0.00% |  |  |  |
| [11.0-12.0) | 0.00% | 0.00% | 0.00% | [11.0-12.0) | 0.00% | 0.00% | 0.00% | [11.0-12.0) | 0.00% | 0.00% | 0.00% |  |  |  |
| [12.0-13.0) | 0.00% | 0.00% | 0.00% | [12.0-13.0) | 0.00% | 0.00% | 0.00% | [12.0-13.0) | 0.00% | 0.00% | 0.00% |  |  |  |
| [13.0-14.0) | 6.67% | 6.67% | 3.33% | [13.0-14.0) | 0.00% | 0.00% | 0.00% | [13.0-14.0) | 7.14% | 7.14% | 3.57% |  |  |  |
| [14.0-15.0) | 10.26% | 10.26% | 7.69% | [14.0-15.0) | 0.00% | 0.00% | 0.00% | [14.0-15.0) | 10.81% | 10.81% | 8.11% |  |  |  |
| [15.0-16.0) | 9.52% | 9.52% | 7.14% | [15.0-16.0) | 0.00% | 0.00% | 0.00% | [15.0-16.0) | 10.00% | 10.00% | 7.50% |  |  |  |
| [16.0-17.0) | 11.32% | 11.32% | 7.55% | [16.0-17.0) | 33.33% | 33.33% | 33.33% | [16.0-17.0) | 10.00% | 10.00% | 6.00% |  |  |  |
| [17.0-18.0) | 9.68% | 9.68% | 6.45% | [17.0-18.0) | 25.00% | 25.00% | 25.00% | [17.0-18.0) | 8.62% | 8.62% | 5.17% |  |  |  |
| [18.0-19.0) | 12.99% | 12.99% | 7.79% | [18.0-19.0) | 28.57% | 28.57% | 28.57% | [18.0-19.0) | 11.43% | 11.43% | 5.71% |  |  |  |
| [19.0-20.0) | 14.29% | 14.29% | 9.52% | [19.0-20.0) | 28.57% | 28.57% | 28.57% | [19.0-20.0) | 12.99% | 12.99% | 7.79% |  |  |  |
| [20.0-21.0) | 16.67% | 16.67% | 10.42% | [20.0-21.0) | 37.50% | 37.50% | 25.00% | [20.0-21.0) | 14.77% | 14.77% | 9.09% |  |  |  |
| [21.0-22.0) | 23.28% | 22.41% | 16.38% | [21.0-22.0) | 53.85% | 46.15% | 46.15% | [21.0-22.0) | 19.42% | 19.42% | 12.62% |  |  |  |
| [22.0-23.0) | 25.38% | 23.08% | 18.46% | [22.0-23.0) | 57.14% | 42.86% | 50.00% | [22.0-23.0) | 21.55% | 20.69% | 14.66% |  |  |  |
| [23.0-24.0) | 26.32% | 23.68% | 18.42% | [23.0-24.0) | 58.82% | 41.18% | 52.94% | [23.0-24.0) | 22.22% | 21.48% | 14.07% |  |  |  |
| [24.0-25.0) | 28.65% | 25.73% | 20.47% | [24.0-25.0) | 61.11% | 44.44% | 55.56% | [24.0-25.0) | 24.84% | 23.53% | 16.34% |  |  |  |
| [25.0-26.0) | 28.81% | 25.99% | 20.90% | [25.0-26.0) | 61.11% | 44.44% | 55.56% | [25.0-26.0) | 25.16% | 23.90% | 16.98% |  |  |  |
| [26.0-27.0) | 30.57% | 27.46% | 22.28% | [26.0-27.0) | 56.00% | 44.00% | 52.00% | [26.0-27.0) | 26.79% | 25.00% | 17.86% |  |  |  |
| [27.0-28.0) | 33.01% | 29.13% | 23.79% | [27.0-28.0) | 56.00% | 44.00% | 52.00% | [27.0-28.0) | 29.83% | 27.07% | 19.89% |  |  |  |
| [28.0-29.0) | 33.48% | 29.07% | 23.79% | [28.0-29.0) | 57.69% | 42.31% | 53.85% | [28.0-29.0) | 30.35% | 27.36% | 19.90% |  |  |  |
| [29.0-30.0] | 34.01% | 29.15% | 24.70% | [29.0-30.0] | 60.00% | 46.67% | 53.33% | [29.0-30.0] | 30.41% | 26.73% | 20.74% |  |  |  |

| **Supplement Table 12: The detection rates of lymph node metastasis in T1-category lung cancer patients with EGFR mutation (N1-N3).** | | | | | | | | | | | | | |
| --- | --- | --- | --- | --- | --- | --- | --- | --- | --- | --- | --- | --- | --- |
|  | **All** | | |  |  | **Solid** | | |  |  | **Sub-solid** | | |
| Diameter (mm) | N1 | N2 | N3 |  |  | N1 | N2 | N3 |  |  | N1 | N2 | N3 |
| [5.0-6.0) | 0.00% | 0.00% | 0.00% |  | [5.0-6.0) | 0.00% | 0.00% | 0.00% |  | [5.0-6.0) | 0.00% | 0.00% | 0.00% |
| [6.0-7.0) | 0.00% | 0.00% | 0.00% |  | [6.0-7.0) | 0.00% | 0.00% | 0.00% |  | [6.0-7.0) | 0.00% | 0.00% | 0.00% |
| [7.0-8.0) | 0.00% | 0.00% | 0.00% |  | [7.0-8.0) | 0.00% | 0.00% | 0.00% |  | [7.0-8.0) | 0.00% | 0.00% | 0.00% |
| [8.0-9.0) | 0.00% | 0.00% | 0.00% |  | [8.0-9.0) | 0.00% | 0.00% | 0.00% |  | [8.0-9.0) | 0.00% | 0.00% | 0.00% |
| [9.0-10.0) | 0.00% | 0.00% | 0.00% |  | [9.0-10.0) | 0.00% | 0.00% | 0.00% |  | [9.0-10.0) | 0.00% | 0.00% | 0.00% |
| [10.0-11.0) | 0.00% | 0.00% | 0.00% |  | [10.0-11.0) | 0.00% | 0.00% | 0.00% |  | [10.0-11.0) | 0.00% | 0.00% | 0.00% |
| [11.0-12.0) | 0.00% | 0.00% | 0.00% |  | [11.0-12.0) | 0.00% | 0.00% | 0.00% |  | [11.0-12.0) | 0.00% | 0.00% | 0.00% |
| [12.0-13.0) | 0.00% | 0.00% | 0.00% |  | [12.0-13.0) | 0.00% | 0.00% | 0.00% |  | [12.0-13.0) | 0.00% | 0.00% | 0.00% |
| [13.0-14.0) | 6.67% | 6.67% | 0.00% |  | [13.0-14.0) | 0.00% | 0.00% | 0.00% |  | [13.0-14.0) | 7.14% | 7.14% | 0.00% |
| [14.0-15.0) | 10.26% | 10.26% | 5.13% |  | [14.0-15.0) | 0.00% | 0.00% | 0.00% |  | [14.0-15.0) | 10.81% | 10.81% | 5.41% |
| [15.0-16.0) | 9.52% | 9.52% | 4.76% |  | [15.0-16.0) | 0.00% | 0.00% | 0.00% |  | [15.0-16.0) | 10.00% | 10.00% | 5.00% |
| [16.0-17.0) | 11.32% | 9.43% | 5.66% |  | [16.0-17.0) | 33.33% | 33.33% | 33.33% |  | [16.0-17.0) | 10.00% | 8.00% | 4.00% |
| [17.0-18.0) | 9.68% | 8.06% | 4.84% |  | [17.0-18.0) | 25.00% | 25.00% | 25.00% |  | [17.0-18.0) | 8.62% | 6.90% | 3.45% |
| [18.0-19.0) | 11.69% | 11.69% | 7.79% |  | [18.0-19.0) | 28.57% | 28.57% | 28.57% |  | [18.0-19.0) | 10.00% | 10.00% | 5.71% |
| [19.0-20.0) | 13.10% | 13.10% | 9.52% |  | [19.0-20.0) | 28.57% | 28.57% | 28.57% |  | [19.0-20.0) | 11.69% | 11.69% | 7.79% |
| [20.0-21.0) | 15.63% | 14.58% | 10.42% |  | [20.0-21.0) | 37.50% | 37.50% | 37.50% |  | [20.0-21.0) | 13.64% | 12.50% | 7.95% |
| [21.0-22.0) | 20.69% | 17.24% | 9.48% |  | [21.0-22.0) | 46.15% | 38.46% | 30.77% |  | [21.0-22.0) | 17.48% | 14.56% | 6.80% |
| [22.0-23.0) | 21.54% | 18.46% | 10.00% |  | [22.0-23.0) | 42.86% | 35.71% | 28.57% |  | [22.0-23.0) | 18.97% | 16.38% | 7.76% |
| [23.0-24.0) | 22.37% | 19.74% | 11.18% |  | [23.0-24.0) | 41.18% | 35.29% | 29.41% |  | [23.0-24.0) | 20.00% | 17.78% | 8.89% |
| [24.0-25.0) | 23.98% | 22.22% | 12.28% |  | [24.0-25.0) | 44.44% | 38.89% | 27.78% |  | [24.0-25.0) | 21.57% | 20.26% | 10.46% |
| [25.0-26.0) | 24.29% | 22.03% | 12.43% |  | [25.0-26.0) | 44.44% | 38.89% | 27.78% |  | [25.0-26.0) | 22.01% | 20.13% | 10.69% |
| [26.0-27.0) | 25.91% | 23.83% | 13.47% |  | [26.0-27.0) | 44.00% | 40.00% | 32.00% |  | [26.0-27.0) | 23.21% | 21.43% | 10.71% |
| [27.0-28.0) | 27.18% | 25.73% | 14.08% |  | [27.0-28.0) | 44.00% | 40.00% | 32.00% |  | [27.0-28.0) | 24.86% | 23.76% | 11.60% |
| [28.0-29.0) | 26.87% | 25.55% | 14.54% |  | [28.0-29.0) | 42.31% | 38.46% | 30.77% |  | [28.0-29.0) | 24.88% | 23.88% | 12.44% |
| [29.0-30.0] | 26.72% | 25.51% | 14.57% |  | [29.0-30.0] | 46.67% | 40.00% | 30.00% |  | [29.0-30.0] | 23.96% | 23.50% | 12.44% |

| **Supplement Table 13: The detection rates of distant metastasis in T1-category lung cancer patients with EGFR mutation (M1a-M1c).** | | | | | | | | | | | | | |
| --- | --- | --- | --- | --- | --- | --- | --- | --- | --- | --- | --- | --- | --- |
|  | **All** | | |  |  | **Solid** | | |  |  | **Sub-solid** | | |
| Diameter (mm) | M1a | M1b | M1c |  |  | M1a | M1b | M1c |  |  | M1a | M1b | M1c |
| [5.0-6.0) | 0.00% | 0.00% | 0.00% |  | [5.0-6.0) | 0.00% | 0.00% | 0.00% |  | [5.0-6.0) | 0.00% | 0.00% | 0.00% |
| [6.0-7.0) | 0.00% | 0.00% | 0.00% |  | [6.0-7.0) | 0.00% | 0.00% | 0.00% |  | [6.0-7.0) | 0.00% | 0.00% | 0.00% |
| [7.0-8.0) | 0.00% | 0.00% | 0.00% |  | [7.0-8.0) | 0.00% | 0.00% | 0.00% |  | [7.0-8.0) | 0.00% | 0.00% | 0.00% |
| [8.0-9.0) | 0.00% | 0.00% | 0.00% |  | [8.0-9.0) | 0.00% | 0.00% | 0.00% |  | [8.0-9.0) | 0.00% | 0.00% | 0.00% |
| [9.0-10.0) | 0.00% | 0.00% | 0.00% |  | [9.0-10.0) | 0.00% | 0.00% | 0.00% |  | [9.0-10.0) | 0.00% | 0.00% | 0.00% |
| [10.0-11.0) | 0.00% | 0.00% | 0.00% |  | [10.0-11.0) | 0.00% | 0.00% | 0.00% |  | [10.0-11.0) | 0.00% | 0.00% | 0.00% |
| [11.0-12.0) | 0.00% | 0.00% | 0.00% |  | [11.0-12.0) | 0.00% | 0.00% | 0.00% |  | [11.0-12.0) | 0.00% | 0.00% | 0.00% |
| [12.0-13.0) | 0.00% | 0.00% | 0.00% |  | [12.0-13.0) | 0.00% | 0.00% | 0.00% |  | [12.0-13.0) | 0.00% | 0.00% | 0.00% |
| [13.0-14.0) | 0.00% | 3.33% | 3.33% |  | [13.0-14.0) | 0.00% | 0.00% | 0.00% |  | [13.0-14.0) | 0.00% | 3.57% | 3.57% |
| [14.0-15.0) | 2.56% | 5.13% | 5.13% |  | [14.0-15.0) | 0.00% | 0.00% | 0.00% |  | [14.0-15.0) | 2.70% | 5.41% | 5.41% |
| [15.0-16.0) | 2.38% | 4.76% | 4.76% |  | [15.0-16.0) | 0.00% | 0.00% | 0.00% |  | [15.0-16.0) | 2.50% | 5.00% | 5.00% |
| [16.0-17.0) | 3.77% | 5.66% | 5.66% |  | [16.0-17.0) | 33.33% | 33.33% | 33.33% |  | [16.0-17.0) | 2.00% | 4.00% | 4.00% |
| [17.0-18.0) | 3.23% | 4.84% | 4.84% |  | [17.0-18.0) | 25.00% | 25.00% | 25.00% |  | [17.0-18.0) | 1.72% | 3.45% | 3.45% |
| [18.0-19.0) | 5.19% | 6.49% | 6.49% |  | [18.0-19.0) | 28.57% | 28.57% | 28.57% |  | [18.0-19.0) | 2.86% | 4.29% | 4.29% |
| [19.0-20.0) | 7.14% | 8.33% | 7.14% |  | [19.0-20.0) | 28.57% | 28.57% | 28.57% |  | [19.0-20.0) | 5.19% | 6.49% | 5.19% |
| [20.0-21.0) | 6.25% | 9.38% | 8.33% |  | [20.0-21.0) | 25.00% | 25.00% | 25.00% |  | [20.0-21.0) | 4.55% | 7.95% | 6.82% |
| [21.0-22.0) | 12.07% | 10.34% | 8.62% |  | [21.0-22.0) | 46.15% | 15.38% | 15.38% |  | [21.0-22.0) | 7.77% | 9.71% | 7.77% |
| [22.0-23.0) | 13.85% | 12.31% | 9.23% |  | [22.0-23.0) | 50.00% | 21.43% | 21.43% |  | [22.0-23.0) | 9.48% | 11.21% | 7.76% |
| [23.0-24.0) | 14.47% | 11.84% | 9.21% |  | [23.0-24.0) | 52.94% | 23.53% | 23.53% |  | [23.0-24.0) | 9.63% | 10.37% | 7.41% |
| [24.0-25.0) | 15.79% | 13.45% | 9.94% |  | [24.0-25.0) | 50.00% | 27.78% | 27.78% |  | [24.0-25.0) | 11.76% | 11.76% | 7.84% |
| [25.0-26.0) | 15.82% | 14.12% | 10.73% |  | [25.0-26.0) | 50.00% | 27.78% | 27.78% |  | [25.0-26.0) | 11.95% | 12.58% | 8.81% |
| [26.0-27.0) | 18.13% | 14.51% | 11.40% |  | [26.0-27.0) | 48.00% | 32.00% | 32.00% |  | [26.0-27.0) | 13.69% | 11.90% | 8.33% |
| [27.0-28.0) | 18.45% | 15.53% | 12.14% |  | [27.0-28.0) | 48.00% | 32.00% | 32.00% |  | [27.0-28.0) | 14.36% | 13.26% | 9.39% |
| [28.0-29.0) | 18.50% | 15.42% | 12.33% |  | [28.0-29.0) | 50.00% | 30.77% | 30.77% |  | [28.0-29.0) | 14.43% | 13.43% | 9.95% |
| [29.0-30.0] | 19.43% | 15.79% | 12.96% |  | [29.0-30.0] | 50.00% | 30.00% | 30.00% |  | [29.0-30.0] | 15.21% | 13.82% | 10.60% |

| **Supplement Table 14: The detection rates of metastasis in T1-category lung cancer patients with EGFR wild-type (overall metastasis, lymph node metastasis and distant metastasis).** | | | | | | | | | | | |  |  |
| --- | --- | --- | --- | --- | --- | --- | --- | --- | --- | --- | --- | --- | --- |
|  | **All** | | |  | **Solid** | | |  | **Sub-solid** | | |  |  |
| Diameter (mm) | Overall metastasis | Lymph node metastasis | Distant metastasis |  | Overall metastasis | Lymph node metastasis | Distant metastasis |  | Overall metastasis | Lymph node metastasis | Distant metastasis |  |  |
| [5.0-6.0) | 0.00% | 0.00% | 0.00% | [5.0-6.0) | 0.00% | 0.00% | 0.00% | [5.0-6.0) | 0.00% | 0.00% | 0.00% |  |  |
| [6.0-7.0) | 0.00% | 0.00% | 0.00% | [6.0-7.0) | 0.00% | 0.00% | 0.00% | [6.0-7.0) | 0.00% | 0.00% | 0.00% |  |  |
| [7.0-8.0) | 0.00% | 0.00% | 0.00% | [7.0-8.0) | 0.00% | 0.00% | 0.00% | [7.0-8.0) | 0.00% | 0.00% | 0.00% |  |  |
| [8.0-9.0) | 0.00% | 0.00% | 0.00% | [8.0-9.0) | 0.00% | 0.00% | 0.00% | [8.0-9.0) | 0.00% | 0.00% | 0.00% |  |  |
| [9.0-10.0) | 0.00% | 0.00% | 0.00% | [9.0-10.0) | 0.00% | 0.00% | 0.00% | [9.0-10.0) | 0.00% | 0.00% | 0.00% |  |  |
| [10.0-11.0) | 5.88% | 0.00% | 5.88% | [10.0-11.0) | 0.00% | 0.00% | 0.00% | [10.0-11.0) | 6.25% | 0.00% | 6.25% |  |  |
| [11.0-12.0) | 7.41% | 3.70% | 7.41% | [11.0-12.0) | 50.00% | 50.00% | 50.00% | [11.0-12.0) | 4.00% | 0.00% | 4.00% |  |  |
| [12.0-13.0) | 13.89% | 11.11% | 8.33% | [12.0-13.0) | 50.00% | 50.00% | 50.00% | [12.0-13.0) | 11.76% | 8.82% | 5.88% |  |  |
| [13.0-14.0) | 12.24% | 10.20% | 8.16% | [13.0-14.0) | 33.33% | 33.33% | 33.33% | [13.0-14.0) | 10.87% | 8.70% | 6.52% |  |  |
| [14.0-15.0) | 10.34% | 8.62% | 6.90% | [14.0-15.0) | 25.00% | 25.00% | 25.00% | [14.0-15.0) | 9.26% | 7.41% | 5.56% |  |  |
| [15.0-16.0) | 12.12% | 9.09% | 9.09% | [15.0-16.0) | 16.67% | 16.67% | 16.67% | [15.0-16.0) | 11.67% | 8.33% | 8.33% |  |  |
| [16.0-17.0) | 13.10% | 10.71% | 7.14% | [16.0-17.0) | 11.11% | 11.11% | 11.11% | [16.0-17.0) | 13.33% | 10.67% | 6.67% |  |  |
| [17.0-18.0) | 16.48% | 14.29% | 9.89% | [17.0-18.0) | 20.00% | 20.00% | 20.00% | [17.0-18.0) | 16.05% | 13.58% | 8.64% |  |  |
| [18.0-19.0) | 17.48% | 15.53% | 9.71% | [18.0-19.0) | 20.00% | 20.00% | 20.00% | [18.0-19.0) | 17.20% | 15.05% | 8.60% |  |  |
| [19.0-20.0) | 19.09% | 15.45% | 10.91% | [19.0-20.0) | 20.00% | 20.00% | 20.00% | [19.0-20.0) | 19.00% | 15.00% | 10.00% |  |  |
| [20.0-21.0) | 20.83% | 16.67% | 13.33% | [20.0-21.0) | 18.18% | 18.18% | 18.18% | [20.0-21.0) | 21.10% | 16.51% | 12.84% |  |  |
| [21.0-22.0) | 26.76% | 21.83% | 19.01% | [21.0-22.0) | 29.41% | 23.53% | 23.53% | [21.0-22.0) | 26.40% | 21.60% | 18.40% |  |  |
| [22.0-23.0) | 29.56% | 23.90% | 20.75% | [22.0-23.0) | 28.57% | 23.81% | 19.05% | [22.0-23.0) | 29.71% | 23.91% | 21.01% |  |  |
| [23.0-24.0) | 32.96% | 27.93% | 22.35% | [23.0-24.0) | 36.00% | 32.00% | 20.00% | [23.0-24.0) | 32.47% | 27.27% | 22.73% |  |  |
| [24.0-25.0) | 35.90% | 30.26% | 22.56% | [24.0-25.0) | 46.88% | 37.50% | 28.13% | [24.0-25.0) | 33.74% | 28.83% | 21.47% |  |  |
| [25.0-26.0) | 37.02% | 31.25% | 22.12% | [25.0-26.0) | 45.95% | 35.14% | 27.03% | [25.0-26.0) | 35.09% | 30.41% | 21.05% |  |  |
| [26.0-27.0) | 38.50% | 30.97% | 22.12% | [26.0-27.0) | 50.00% | 35.00% | 27.50% | [26.0-27.0) | 36.02% | 30.11% | 20.97% |  |  |
| [27.0-28.0) | 40.24% | 32.11% | 22.76% | [27.0-28.0) | 53.49% | 37.21% | 32.56% | [27.0-28.0) | 37.44% | 31.03% | 20.69% |  |  |
| [28.0-29.0) | 40.77% | 32.31% | 22.31% | [28.0-29.0) | 53.33% | 37.78% | 31.11% | [28.0-29.0) | 38.14% | 31.16% | 20.47% |  |  |
| [29.0-30.0] | 41.13% | 32.98% | 22.70% | [29.0-30.0] | 54.17% | 37.50% | 29.17% | [29.0-30.0] | 38.46% | 32.05% | 21.37% |  |  |

| **Supplement Table 15: The detection rates of lymph node metastasis in T1-category lung cancer patients with EGFR wild-type (N1-N3).** | | | | | | | | | | | | | |
| --- | --- | --- | --- | --- | --- | --- | --- | --- | --- | --- | --- | --- | --- |
|  | **All** | | |  |  | **Solid** | | |  |  | **Sub-solid** | | |
| Diameter (mm) | N1 | N2 | N3 |  |  | N1 | N2 | N3 |  |  | N1 | N2 | N3 |
| [5.0-6.0) | 0.00% | 0.00% | 0.00% |  | [5.0-6.0) | 0.00% | 0.00% | 0.00% |  | [5.0-6.0) | 0.00% | 0.00% | 0.00% |
| [6.0-7.0) | 0.00% | 0.00% | 0.00% |  | [6.0-7.0) | 0.00% | 0.00% | 0.00% |  | [6.0-7.0) | 0.00% | 0.00% | 0.00% |
| [7.0-8.0) | 0.00% | 0.00% | 0.00% |  | [7.0-8.0) | 0.00% | 0.00% | 0.00% |  | [7.0-8.0) | 0.00% | 0.00% | 0.00% |
| [8.0-9.0) | 0.00% | 0.00% | 0.00% |  | [8.0-9.0) | 0.00% | 0.00% | 0.00% |  | [8.0-9.0) | 0.00% | 0.00% | 0.00% |
| [9.0-10.0) | 0.00% | 0.00% | 0.00% |  | [9.0-10.0) | 0.00% | 0.00% | 0.00% |  | [9.0-10.0) | 0.00% | 0.00% | 0.00% |
| [10.0-11.0) | 0.00% | 0.00% | 0.00% |  | [10.0-11.0) | 0.00% | 0.00% | 0.00% |  | [10.0-11.0) | 0.00% | 0.00% | 0.00% |
| [11.0-12.0) | 3.70% | 3.70% | 3.70% |  | [11.0-12.0) | 50.00% | 50.00% | 50.00% |  | [11.0-12.0) | 0.00% | 0.00% | 0.00% |
| [12.0-13.0) | 11.11% | 11.11% | 8.33% |  | [12.0-13.0) | 50.00% | 50.00% | 50.00% |  | [12.0-13.0) | 8.82% | 8.82% | 5.88% |
| [13.0-14.0) | 10.20% | 10.20% | 8.16% |  | [13.0-14.0) | 33.33% | 33.33% | 33.33% |  | [13.0-14.0) | 8.70% | 8.70% | 6.52% |
| [14.0-15.0) | 8.62% | 8.62% | 6.90% |  | [14.0-15.0) | 25.00% | 25.00% | 25.00% |  | [14.0-15.0) | 7.41% | 7.41% | 5.56% |
| [15.0-16.0) | 9.09% | 9.09% | 7.58% |  | [15.0-16.0) | 16.67% | 16.67% | 16.67% |  | [15.0-16.0) | 8.33% | 8.33% | 6.67% |
| [16.0-17.0) | 9.52% | 10.71% | 8.33% |  | [16.0-17.0) | 11.11% | 11.11% | 11.11% |  | [16.0-17.0) | 9.33% | 10.67% | 8.00% |
| [17.0-18.0) | 13.19% | 13.19% | 9.89% |  | [17.0-18.0) | 20.00% | 20.00% | 20.00% |  | [17.0-18.0) | 12.35% | 12.35% | 8.64% |
| [18.0-19.0) | 13.59% | 14.56% | 11.65% |  | [18.0-19.0) | 20.00% | 20.00% | 20.00% |  | [18.0-19.0) | 12.90% | 13.98% | 10.75% |
| [19.0-20.0) | 13.64% | 14.55% | 11.82% |  | [19.0-20.0) | 20.00% | 20.00% | 20.00% |  | [19.0-20.0) | 13.00% | 14.00% | 11.00% |
| [20.0-21.0) | 15.00% | 15.83% | 12.50% |  | [20.0-21.0) | 18.18% | 18.18% | 18.18% |  | [20.0-21.0) | 14.68% | 15.60% | 11.93% |
| [21.0-22.0) | 20.42% | 20.42% | 13.38% |  | [21.0-22.0) | 17.65% | 23.53% | 11.76% |  | [21.0-22.0) | 20.80% | 20.00% | 13.60% |
| [22.0-23.0) | 22.01% | 22.64% | 14.47% |  | [22.0-23.0) | 19.05% | 23.81% | 14.29% |  | [22.0-23.0) | 22.46% | 22.46% | 14.49% |
| [23.0-24.0) | 25.14% | 25.70% | 13.97% |  | [23.0-24.0) | 28.00% | 28.00% | 16.00% |  | [23.0-24.0) | 24.68% | 25.32% | 13.64% |
| [24.0-25.0) | 26.67% | 28.21% | 13.85% |  | [24.0-25.0) | 31.25% | 34.38% | 18.75% |  | [24.0-25.0) | 25.77% | 26.99% | 12.88% |
| [25.0-26.0) | 27.40% | 29.33% | 13.46% |  | [25.0-26.0) | 29.73% | 32.43% | 16.22% |  | [25.0-26.0) | 26.90% | 28.65% | 12.87% |
| [26.0-27.0) | 27.88% | 28.76% | 13.72% |  | [26.0-27.0) | 32.50% | 35.00% | 17.50% |  | [26.0-27.0) | 26.88% | 27.42% | 12.90% |
| [27.0-28.0) | 29.67% | 30.49% | 15.04% |  | [27.0-28.0) | 34.88% | 37.21% | 20.93% |  | [27.0-28.0) | 28.57% | 29.06% | 13.79% |
| [28.0-29.0) | 29.62% | 30.38% | 15.00% |  | [28.0-29.0) | 35.56% | 35.56% | 20.00% |  | [28.0-29.0) | 28.37% | 29.30% | 13.95% |
| [29.0-30.0] | 30.50% | 30.85% | 15.60% |  | [29.0-30.0] | 37.50% | 33.33% | 18.75% |  | [29.0-30.0] | 29.06% | 30.34% | 14.96% |

| **Supplement Table 16: The detection rates of distant metastasis in T1-category lung cancer patients with EGFR wild-type (M1a-M1c).** | | | | | | | | | | | | | |
| --- | --- | --- | --- | --- | --- | --- | --- | --- | --- | --- | --- | --- | --- |
|  | **All** | | |  |  | **Solid** | | |  |  | **Sub-solid** | | |
| Diameter (mm) | M1a | M1b | M1c |  |  | M1a | M1b | M1c |  |  | M1a | M1b | M1c |
| [5.0-6.0) | 0.00% | 0.00% | 0.00% |  | [5.0-6.0) | 0.00% | 0.00% | 0.00% |  | [5.0-6.0) | 0.00% | 0.00% | 0.00% |
| [6.0-7.0) | 0.00% | 0.00% | 0.00% |  | [6.0-7.0) | 0.00% | 0.00% | 0.00% |  | [6.0-7.0) | 0.00% | 0.00% | 0.00% |
| [7.0-8.0) | 0.00% | 0.00% | 0.00% |  | [7.0-8.0) | 0.00% | 0.00% | 0.00% |  | [7.0-8.0) | 0.00% | 0.00% | 0.00% |
| [8.0-9.0) | 0.00% | 0.00% | 0.00% |  | [8.0-9.0) | 0.00% | 0.00% | 0.00% |  | [8.0-9.0) | 0.00% | 0.00% | 0.00% |
| [9.0-10.0) | 0.00% | 0.00% | 0.00% |  | [9.0-10.0) | 0.00% | 0.00% | 0.00% |  | [9.0-10.0) | 0.00% | 0.00% | 0.00% |
| [10.0-11.0) | 0.00% | 5.88% | 5.88% |  | [10.0-11.0) | 0.00% | 0.00% | 0.00% |  | [10.0-11.0) | 0.00% | 6.25% | 6.25% |
| [11.0-12.0) | 0.00% | 7.41% | 3.70% |  | [11.0-12.0) | 0.00% | 50.00% | 0.00% |  | [11.0-12.0) | 0.00% | 4.00% | 4.00% |
| [12.0-13.0) | 2.78% | 8.33% | 5.56% |  | [12.0-13.0) | 0.00% | 50.00% | 0.00% |  | [12.0-13.0) | 2.94% | 5.88% | 5.88% |
| [13.0-14.0) | 2.04% | 8.16% | 6.12% |  | [13.0-14.0) | 0.00% | 33.33% | 0.00% |  | [13.0-14.0) | 2.17% | 6.52% | 6.52% |
| [14.0-15.0) | 1.72% | 6.90% | 5.17% |  | [14.0-15.0) | 0.00% | 25.00% | 0.00% |  | [14.0-15.0) | 1.85% | 5.56% | 5.56% |
| [15.0-16.0) | 1.52% | 9.09% | 6.06% |  | [15.0-16.0) | 0.00% | 16.67% | 0.00% |  | [15.0-16.0) | 1.67% | 8.33% | 6.67% |
| [16.0-17.0) | 1.19% | 7.14% | 4.76% |  | [16.0-17.0) | 0.00% | 11.11% | 0.00% |  | [16.0-17.0) | 1.33% | 6.67% | 5.33% |
| [17.0-18.0) | 1.10% | 9.89% | 7.69% |  | [17.0-18.0) | 0.00% | 20.00% | 10.00% |  | [17.0-18.0) | 1.23% | 8.64% | 7.41% |
| [18.0-19.0) | 1.94% | 8.74% | 6.80% |  | [18.0-19.0) | 0.00% | 20.00% | 10.00% |  | [18.0-19.0) | 2.15% | 7.53% | 6.45% |
| [19.0-20.0) | 2.73% | 9.09% | 7.27% |  | [19.0-20.0) | 0.00% | 20.00% | 10.00% |  | [19.0-20.0) | 3.00% | 8.00% | 7.00% |
| [20.0-21.0) | 5.00% | 10.83% | 9.17% |  | [20.0-21.0) | 0.00% | 18.18% | 9.09% |  | [20.0-21.0) | 5.50% | 10.09% | 9.17% |
| [21.0-22.0) | 11.27% | 14.08% | 11.97% |  | [21.0-22.0) | 11.76% | 17.65% | 11.76% |  | [21.0-22.0) | 11.20% | 13.60% | 12.00% |
| [22.0-23.0) | 13.21% | 15.72% | 11.95% |  | [22.0-23.0) | 9.52% | 14.29% | 9.52% |  | [22.0-23.0) | 13.77% | 15.94% | 12.32% |
| [23.0-24.0) | 14.53% | 16.76% | 12.29% |  | [23.0-24.0) | 12.00% | 16.00% | 8.00% |  | [23.0-24.0) | 14.94% | 16.88% | 12.99% |
| [24.0-25.0) | 14.87% | 16.92% | 12.82% |  | [24.0-25.0) | 18.75% | 21.88% | 15.63% |  | [24.0-25.0) | 14.11% | 15.95% | 12.27% |
| [25.0-26.0) | 14.90% | 15.87% | 12.02% |  | [25.0-26.0) | 18.92% | 18.92% | 13.51% |  | [25.0-26.0) | 14.04% | 15.20% | 11.70% |
| [26.0-27.0) | 15.49% | 15.04% | 11.06% |  | [26.0-27.0) | 20.00% | 20.00% | 12.50% |  | [26.0-27.0) | 14.52% | 13.98% | 10.75% |
| [27.0-28.0) | 16.26% | 15.45% | 11.38% |  | [27.0-28.0) | 23.26% | 25.58% | 18.60% |  | [27.0-28.0) | 14.78% | 13.30% | 9.85% |
| [28.0-29.0) | 16.15% | 14.62% | 10.77% |  | [28.0-29.0) | 22.22% | 24.44% | 17.78% |  | [28.0-29.0) | 14.88% | 12.56% | 9.30% |
| [29.0-30.0] | 16.31% | 14.54% | 10.99% |  | [29.0-30.0] | 20.83% | 22.92% | 16.67% |  | [29.0-30.0] | 15.38% | 12.82% | 9.83% |
